# Mycotransformation of anthracene by indigenous *Trichoderma lixii* and *Talaromyces pinophilus* isolates: insights into the metabolic pathways, enzyme profiles and acute toxicity

**DOI:** 10.1007/s10532-025-10147-z

**Published:** 2025-06-18

**Authors:** Samson O. Egbewale, Ajit Kumar, Mduduzi P. Mokoena, Ademola O. Olaniran

**Affiliations:** 1 Discipline of Microbiology, University of KwaZulu-Natal (Westville Campus), Durban, 4000 South Africa; 2 Department of Pathology, School of Medicine, University of Limpopo, Private Bag, Sovenga, X11060727 South Africa

**Keywords:** Anthracene, Mycotransformation; toxicity, *Trichoderma lixii*, *Talaromyces pinophilus*

## Abstract

This study focused on the mycotransformation of a very prominent PAH, anthracene, and its acute toxicity reduction by Ascomycete fungi: *Trichoderma lixii* strain FLU1 (*Tl*FLU1) and *Talaromyces pinophilus* strain FLU12 (*Tp*FLU12), indigenously isolated from benzo[b] fluoranthene-enriched activated sludge. The results indicate that both the isolates *Tl*FLU1 and *Tp*FLU12 could tolerate anthracene exposure up to 1000 mg/L, with increased expression of ligninolytic enzymes: Laccase, Lignin peroxidase, and Manganese peroxidase. The mycotransformation of anthracene was observed to be growth-linked and mediated by the expression of the intracellular enzymes as the initial mechanism used by these strains followed by the ligninolytic enzymes with up to 56% and 38% anthracene degradation by *Tl*FLU1 and *Tp*FLU12, respectively, after 24 days with a concomitant change in pH from 5 to 4 (*Tl*FLU1) and 6.2 (*Tp*FLU12). The GC–MS and FTIR analysis of the samples indicate the appearance of metabolic intermediates: 9,10 anthracenedione and benzoic acid in *Tl*FLU1 grown medium, while anthrone and 9,10 anthracenedione were detected in *Tp*FLU12 grown medium. The mycotransformation of the compound followed a first-order kinetic model with an effective concentration (EC_50_) of 262.3–266.1 mg/L, with a toxicity unit (TU) of 0.4% in *Vibrio parahaemolyticus* (6 h exposure) to each intermediate. Results show efficient mycotransformation of anthracene into a non-toxic state by *Tl*FLU1 and *Tp*FLU12.

**Supplementary Information:**

The online version contains supplementary material available at 10.1007/s10532-025-10147-z.

## Introduction

Polycyclic aromatic hydrocarbons (PAHs) are a group of recalcitrant and ubiquitous contaminants released into the environment through natural and anthropogenic processes (Nacher-Mestre et al. 2014). They exist as derivatives of biogenic, petrogenic, and pyrolytic compounds (Baran et al. 2017). Due to the recalcitrant characteristics, hydrophobicity, toxicity, carcinogenic effects, and non-bioavailability to biological agents for degradation, USEPA listed PAHs among priority pollutants that need to be expunged from ecosystems (Souza et al. 2015).

Anthracene, a PAH, is the active ingredient in various products, including dyes, synthetic fibers, and wood preservatives (Ellickson et al. 2020; Rengarajan et al. 2015). These applications pose significant environmental hazards as anthracene acts as an immunosuppressant, teratogen, and respiratory irritant, with multiple degrees of toxicity, mutagenicity, and carcinogenicity. Following its widespread use, anthracene is found in various environmental compartments, with its levels varying significantly. In urban air, its concentration ranges from 0.1 to 10 ng/m^3^; in rural areas, it is lower, at 0.01 to 1 ng/m^3^. However, in cigarette smoke, its levels can be much higher, at 100–1000 ng/m^3^. In soil, its concentrations can reach up to 10,000 µg/kg in areas near industrial zones or coal tar deposits (Yang et al. 1991). The distribution of PAHs in aquatic ecosystems is influenced by particle size and organic matter content (Gao et al. 1998). Their subcooled liquid vapor pressure influences gas-particle partitioning of PAHs in urban, coastal, and continental sites (Terzi and Samara 2004). The concentrations of PAHs in soils are affected by soil properties and climate, with lighter PAHs being more prevalent in the south, suggesting biological sources (Wilcke et al. 2014).

In plants, anthracene accounts for low yields in crops through the interference with carbon fixation inhibition, enzymatic dysfunction, membrane permeability, and reduction in net photosynthesis (Jajoo et al. 2014). Researchers and environmental agencies have been using techniques like chemical oxidation, flocculation, activated carbon, electro-oxidation, and photolysis to mitigate the harmful effects of anthracene (Eeshwarasinghe et al. 2019). However, these techniques are not as efficient as microbial-mediated detoxification/biotransformation methods, which are also deemed to be eco-friendly and a low-cost process (Adnan et al. 2016). For instance, microbial degradation has been shown to achieve higher removal rates under optimal conditions compared to physicochemical methods, as demonstrated in recent studies (Egbewale et al. 2023; Li et al. 2024; Woo et al. 2009). While the experimental period in this study was 24 days, microbial processes often offer long-term sustainability and reduced secondary pollution, making them a preferred choice for large-scale applications.

Mycotransformation by mushroom-forming fungi (Basidiomycetes) has received tremendous attention over the past decades owing to their effective biotransformation potential through aggressive growth and ligninolytic enzyme production (Adenipekun et al. 2015). Mycotransformation of PAHs is primarily mediated via oxidation by forming quinone intermediates involving three enzymes, namely, Laccase (Lac, p-Diphenol: dioxygen oxidoreductases; EC 1.10.3.2), Manganese peroxidase (MnP; EC 1.11.1.13), and Lignin peroxidase (LiP; EC 1.11.1.14) (Bilal et al. 2019). PAHs reportedly induced these enzymes during myco-degradation processes (Bankole et al. 2021; Hadibarata and Kristanti 2012). However, PAHs mycotransformation products, such as dihydrodiol (quinones) and epoxides, are of great environmental concern due to their deleterious effect compared to their parent compounds (Jove et al. 2016; Mtibaà et al. 2018a). The transcriptomic responses of catalase, peroxidase, and laccase encoding genes and the enzymatic activities of oil-spill-inhabiting rhizospheric fungal strains, *Talaromyces purpurogenum* and *Trichoderma harzianum*, showed overexpression of genes encoding lignin peroxidase (LiP) and manganese peroxidase (MnP) in response to crude oil contamination (Asemoloye et al. 2018). The Ascomycete fungi could contain other types of Class II peroxidases like AA, AB, and AC type peroxidases, along with DyP type peroxidases and some other intracellular Class II peroxidases (Hammel et al. 1991; Kimani et al. 2021; Morgenstern et al. 2008; Zámocký et al. 2016; Zeiner et al. 2016).

Ecotoxicity is a crucial tool for environmental safety and protection assessment. Although most ecotoxicity bioassays comprise using representatives from all trophic levels, namely, bacteria, which role is decomposers, algae (producers), invertebrates, and vertebrates as the consumers (Turek et al. 2019). Yet, the choice of conventional bacteria bioluminescence inhibition tests on toxicants from elutriate samples has a lot of drawbacks, ranging from bacterial adsorption on surfaces, interference by sulfur or sulfides, ammonia, interference of bioluminescence due to pH variations, color, or particles (Jarque et al. 2016). Bacteria survival assay using marine bacterium (*Vibrio spp.*) as a bioindicator for ecotoxicity assessment is simple, fast, and cheap, corroborating the other toxicity assay responses (Rotini et al. 2017).

Despite the overwhelming reports on the potential of basidiomycetes in the mycotransformation of PAHs, little literature is available on the mycotransformation of PAHs by non-mushroom forming fungi from the ascomycetes, mechanisms, and pathways for their mycotransformation and ecotoxicity testing of PAHs/metabolites. Thus, this study seeks to bridge the knowledge gaps by determining the tolerance level of the indigenous *Trichoderma lixii* strain FLU1 and *Talaromyces pinophilus* strain FLU12 to a varying concentration of anthracene. Metabolite profiling and characterization of enzymes established the biodegradation pathway. The mycotransformation efficiency, mycotransformation kinetics, and acute toxicity of anthracene on the fungal strains and the transient mycotransformation products were also elucidated in this study.

## Materials and methods

### Materials

All reagents used are of HPLC grade with ≥ 98% purity. Anthracene, ethyl acetate, ABTS, DMP, azure B, catechol, CaCl_2_^.^2H_2_O, (NH_4_)_2_SO_4_, K_2_HPO_4_, MnCl_2_^.^2H_2_O, NaH_2_PO_4_^.^2H_2_O, NaCl, MgSO_4_^.^7H_2_O, Nitrilotriacetic acid, FeSO_4_^.^7H_2_O, ZnSO_4,_ CuSO_4_, H_3_BO_3_, Na_2_MoO_4_, Co(NO_3_)^.^6H_2_O, Al_2_(SO_4_)_3_^.^H_2_O, PDA and TCBS agar were bought from Merck (NJ, USA). 500 mg of anthracene stock solution prepared in 10 mL methanol and sterilized with a 0.22 µm filter (Millipore, Merck, New Jersey, USA). BSM was prepared as described previously (Saraswathy and Hallberg 2002) and pH adjusted to 5 with 1 N HCl. *Tl*FLU1 and *Tp*FLU12 were isolated from benzo[b] fluoranthene-enriched wastewater-activated sludge, and their characteristics with identification have been reported previously (Egbewale et al. 2023).

### Evaluation of anthracene tolerance on PDA plates

Anthracene concentrations of 0, 50, 100, 200, 400, 600, 800, and 1000 mg/L were applied to PDA plates with corresponding concentrations using a sterile rod and left in the laminar hood for 2 h to allow the solvent to evaporate and the anthracene to adhere to the agar surface. Thereafter, mycelia agar plugs (5 mm diameter) from 7-day-old actively growing *Tl*FLU1 and *Tp*FLU12 were separately inoculated at the center of Petri dishes containing anthracene-contaminated PDA plates, incubated at 30 °C for 10 days in four replications under total darkness conditions. Control plates (no anthracene addition) were also included. Radial growth rate was measured for each fungus every 24 h to the end of the incubation period to assess the tolerance of each fungus to varying anthracene concentrations.

The fungal inhibition percentage due to the anthracene exposure was calculated using the following equations:

1 $${\text{FG }}\left( \% \right)\, = \,{\text{D}}_{{{\text{ANT}}}} /{\text{ D}}_{{\text{C}}} \, \times \,{1}00$$

2 $${\text{FI }}\left( \% \right)\, = \,{1}00{-}{\text{FG}}$$

Where FG = Fungal growth rate (FG) was calculated from the radial mycelial growth record.

D_ANT_ = Diameter of fungal colony exposed to anthracene,

DC = Diameter of the control fungal colony (no anthracene addition).

FI = Fungal growth inhibition of the colonies after anthracene exposure at varying concentrations (Argumedo-Delira et al. 2012).

### Qualitative screening of isolated fungi for ligninolytic enzymes

Enzymatic assays for strains *Tl*FLU1 and *Tp*FLU12 were conducted using laccase (Lac), lignin peroxidase (LiP), and manganese peroxidase (MnP) as the target enzymes, as fungi commonly employ them during biodegradation. Lac activity was screened on each anthracene-contaminated PDA plate by adding ABTS (0.1% w/v) as a substrate, and the final pH was adjusted to 5.5. The plates were inoculated at the center with 5 mm mycelial agar plugs from 7-day-old actively growing strains and incubated at 30 °C for 10 days in four replications under total dark conditions. Lac activity was evaluated based on green color development in the presence of ABTS. LiP activity was screened by adding Azure B (0.001% w/v) to the anthracene-contaminated PDA plates and incubating them for 10 days. The reduction in blue/purple color to sky blue indicated LiP activity. MnP activity was screened on Czapek-Dox agar medium containing phenol red (0.0025% w/v). After 10 days of incubation at 30 °C, the reduction in red color to yellow zones indicated MnP activity.

### Anthracene mycotransformation study and fungal strains biomass estimation

The anthracene degradation and fungal biomass estimation studies were conducted as described previously (Hadibarata and Kristanti 2012). Briefly, 400 mg/L anthracene in 100 mL BSM broth was opened in a laminar hood for 20 min to allow the solvent used for anthracene preparation to evaporate to yield a superficial desired concentration before inoculation with mycelium discs of an actively growing culture of each fungal strain. Incubation was allowed for 24 days at 30 °C. Only 400 mg/L anthracene in BSM was used as a negative control. The aliquots at 4-day intervals were withdrawn to quantify residual anthracene, intermediate formed, ligninolytic enzymes activity, and to perform ecotoxicity tests. The initial concentration of 400 mg/L was used based on low fungal growth inhibition compared with higher concentrations on both selected strains (Tables S1 and S2). For fungal biomass estimation, 4 mL culture broth was collected by centrifugation, and the dry biomass of the pellets was determined. Also, the residual anthracene was quantified in the supernatant. Washing the pellet with ethyl acetate (1:10 w/v, 5 times) was necessary to remove the residual anthracene from the pellets, as it could limit biosorption by the fungi mycelia pellets due to its hydrophobic nature.

### Extraction and quantification of residual anthracene

Residual anthracene culture medium was extracted and quantified as previously described (Egbewale et al. 2023; Teerapatsakul et al. 2016) with some modifications (Egbewale et al. 2023). The culture broth supernatant was mixed with 1:4 v/v ethyl acetate, vigorously shaken, and allowed to stand for 20 min. The organic phase was dried and quantified for the residual anthracene. The experiments were triplicated with an average anthracene recovery of 98.7 ± 2.53%. The kinetic models of anthracene mycotransformation were plotted as described previously (Ortega-González et al. 2015). A composite sample from the tetrad days collections of the residual anthracene and intermediates formed during the mycotransformation was also analyzed by GC–MS and FTIR (Egbewale et al. 2023; Teh and Hadibarata 2014).

### Intracellular and extracellular ligninolytic activity assays

The preparation of cell lysate for intracellular activity of catechol 1,2 dioxygenase (C12D, ortho-cleaving) and catechol 2,3 dioxygenase (C23D, meta-cleaving) was carried out as described previously (Mandal and Das 2018). Extracellular activity of Lac (Patel et al. 2009), LiP-like (Agrawal and Shahi 2017) and MnP-like was carried out as described previously (Egbewale et al. 2023). To determine the intracellular activity for Lac, LiP-like, and MnP-like, the fungal biomass was collected after a 4-day interval, filtered, and washed once with BSM. The mycelia were resuspended in 50 mM Na-phosphate buffer (pH 5) and sonicated to get cell-free lysate. The enzyme activities for Lac, LiP-like and MnP-like in the cell lysate were carried out as described previously (Agrawal and Shahi 2017; Egbewale et al. 2023; Patel et al. 2009). One unit of respective enzyme activity was defined as one µmol of *cis,cis*-muconic acid (product) for catechol 1,2 dioxygenase, one µmol of 2-hydroxymuconic semialdehyde (product) for catechol 2,3 dioxygenase, one µmol of ABTS oxidized, one µmol of Azure B reduced, and one µmol of 2,6-DMP oxidized per min at 30 °C, pH 7.

### Ecotoxicity test

The ecotoxicity test was conducted on *Vibrio parahaemolyticus* (ATCC 17802) as described previously (Egbewale et al. 2023). Directive 79/831/EEC (Lin et al. 2004) was used to classify (very toxic, EC_50_ ≤ 1 mg/L; toxic, 1 mg/L ≤ EC_50_ < 10 mg/L; harmful, 10 mg/L ≤ EC_50_ ≤ 100 mg/L and non-toxic, EC_50_ ≥ 100 mg/L).

### Data analysis

A completely randomized design approach was used to set up experiments. Mean values and error bars representing the standard deviation from three independent experiments. Data were analysed using GraphPad Prism version 8.4.3 (v.471, San Diego, CA, USA). The statistical details on EC_50_ and TU can be seen in the legend of Fig. 5.

## Results

### Anthracene mycotransformation, biomass yield, and extracellular protein content and pH in liquid media

**Fig. 1:**
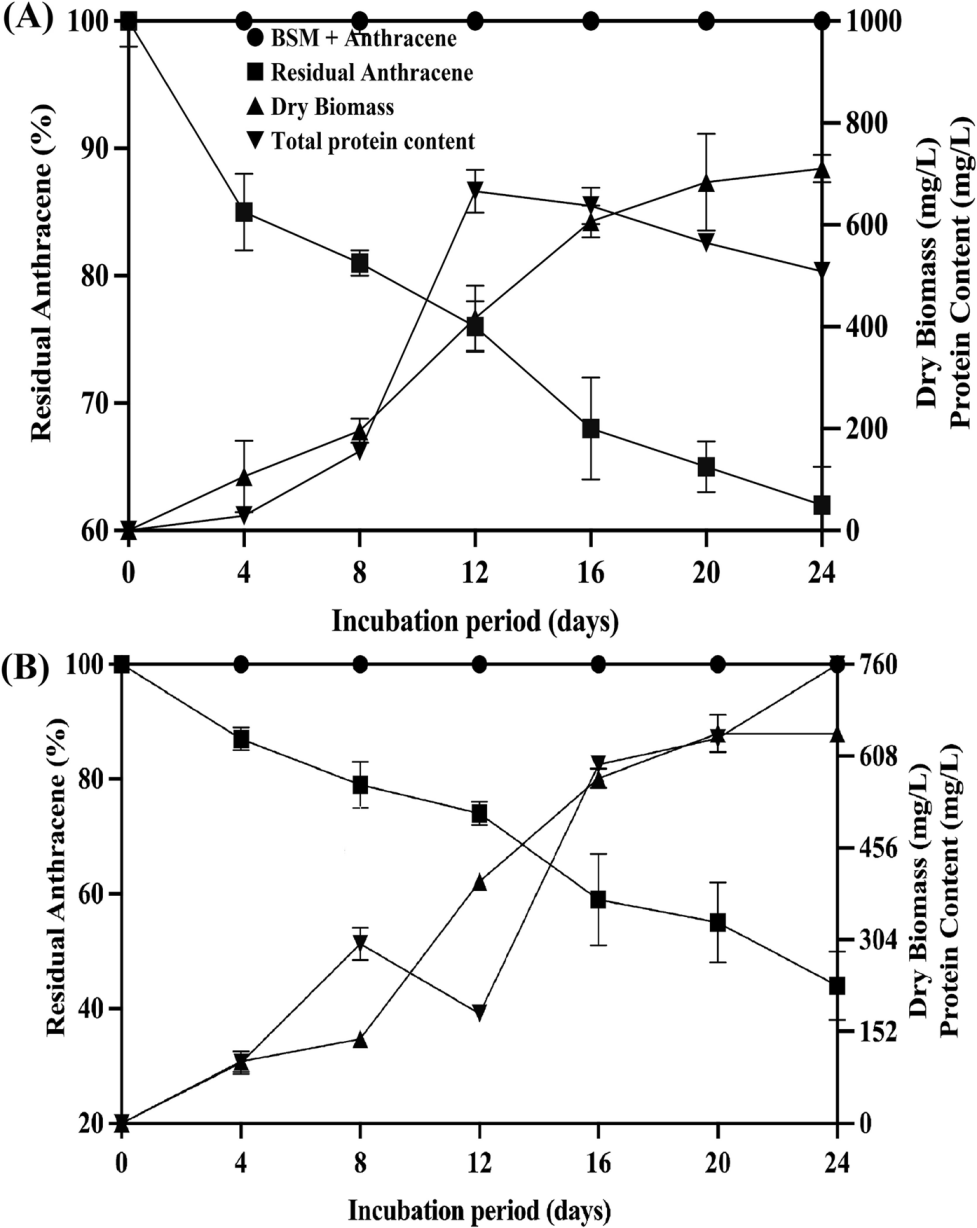
Degradation of anthracene by *Tl*FLU1 **A** and *Tp*FLU12 **B**. Curves shown are BSM + anthracene (●), residual anthracene (■), dry biomass (▲), and total protein content (▼)

**Fig. 2:**
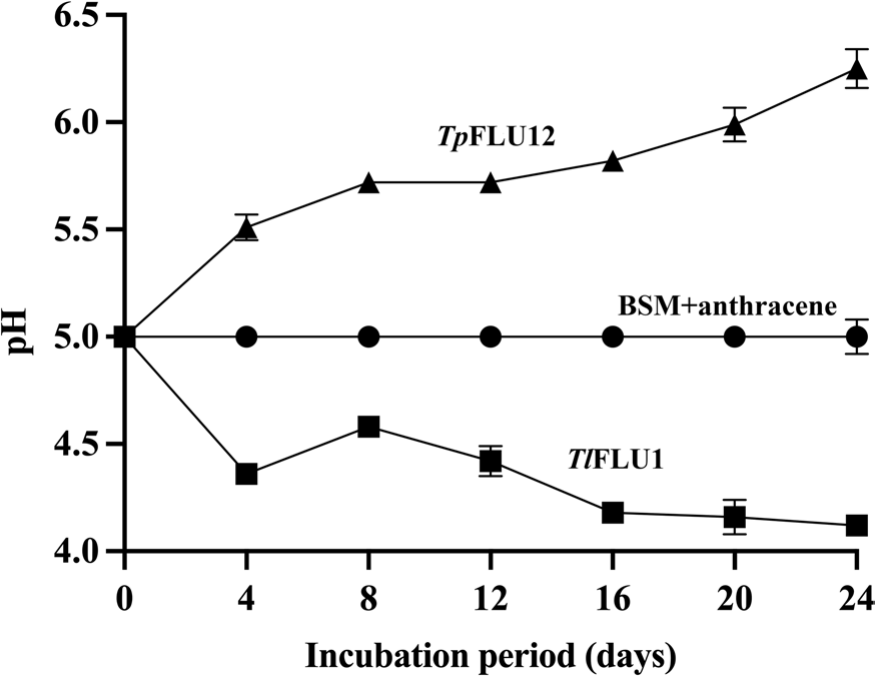
Change in pH of media while anthracene degradation by *Tl*FLU1 (■) and *Tp*FLU12 (▲). BSM + anthracene used as control (●)

The results of anthracene mycotransformation, fungal growth, and extracellular protein content indicate that *Tp*FLU12 transforms anthracene more efficiently than *Tl*FLU1 (Fig. 1). A significant decrease in percentage residual anthracene concentration was observed over time for both fungal strains. In *Tl*FLU1 inoculated flasks, 38% anthracene degradation was observed with dry fungal biomass of 710.3 mg/L at the end of the incubation period of 24 days, while *Tp*FLU12 achieved 56% degradation with a lower biomass of 645.6 mg/L on day 12 of the incubation period (Fig. 1a). The extracellular protein of 760 mg/L was measured on day 24 of the incubation period (Fig. 1b). Furthermore, after the incubation period of 24 days, the flask inoculated with *Tl*FLU1 showed a notable decrease in pH from the initial value of 5.0 to 4.2 (Fig. 2, curve A), while the flask inoculated with *Tp*FLU12 displayed an intriguing increase in pH from the initial 5.0 to 6.25 over the same incubation period (Fig. 2, curve B).

The kinetic parameters of the mycotransformation of anthracene are shown in Table 1 and Fig. S1. The anthracene mycotransformation by strains *Tl*FLU1 and *Tp*FLU12 best fitted the zero-order and first-order kinetics. Under the zero-order kinetics, a rate constant of 5.964 and a half-life of 33.54 with an *R*^2^ value of 0.9402 was obtained for isolate *Tl*FLU1, while the isolate *Tp*FLU12 inoculated flask has a rate constant of 9.000 and a half-life of 22.222 with an *R*^2^ value of 0.9866. However, under the first-order kinetics, *Tp*FLU12 showed a superior rate constant of 0.0328 and a half-life of 21.15 with an *R*^2^ value of 0.9769, while in the *Tl*FLU1 inoculated flask, a rate constant of 0.0192, a half-life of 36.101, and an *R*^2^ value of 0.9670 were obtained. Also, it was observed that both *Tl*FLU1 and *Tp*FLU12 did not obey the second-order kinetics. A constant of 0.0001 and a half-life of 46.668 with *R*^2^ value of 0.7500 was obtained for *Tl*FLU1, while a constant of 0.0001 and a half-life of 20.000 with *R*^2^ value of 0.7903 was obtained for *Tp*FLU12.

**Table 1:** Kinetic parameters for the degradation of anthracene by *Tl*FLU1 and *Tp*FLU12

Kinetic model	Parameter	Strains	
		*Tl*FLU1	*Tp*FLU12
Zero order	Regression Equation	C_d_ =−5.964d + 378.4	C_d_ = -9.000d + 392.6
C_t_—C_0_ = Kt	K(d^−1^)	5.964	9.0000
t_1/2_ = C_0_/2K_0_	t_1/2_	33.535	22.222
	R^2^	0.9402	0.9866
First order	Regression Equation	lnC_d_ =−0.0192d + 5.944	lnC_d_ =−0.0328d + 6.010
lnC_t_ = K_1_t + lnC_0_	K(d^−1^)	0.0192	0.0328
t_1/2_ = ln 2 / K_t_	t_1/2_	36.196	21.145
	R^2^	0.9670	0.9769
Second order	Regression Equation	1/C_d_ = 5.35×10^−5^ + 0.003	1/C_d_ = 0.0001d + 0.002
1/C_t_ = 1/C_0_ + K_2_t	K(d^−1^)	0.0001	0.0001
T_1/2_ = 1/C_0_K_2_	t_1/2_	46.668	20.000
	R^2^	0.7500	0.7903

*R*^2^: Regression coefficient; K: Degradation rate constant; t_1/2_: Half-life period (d); C_0_: Initial concentration; Ct_:_ Final concentration

### Profiles of anthracene mycotransformation metabolic products

**Fig. 3:**
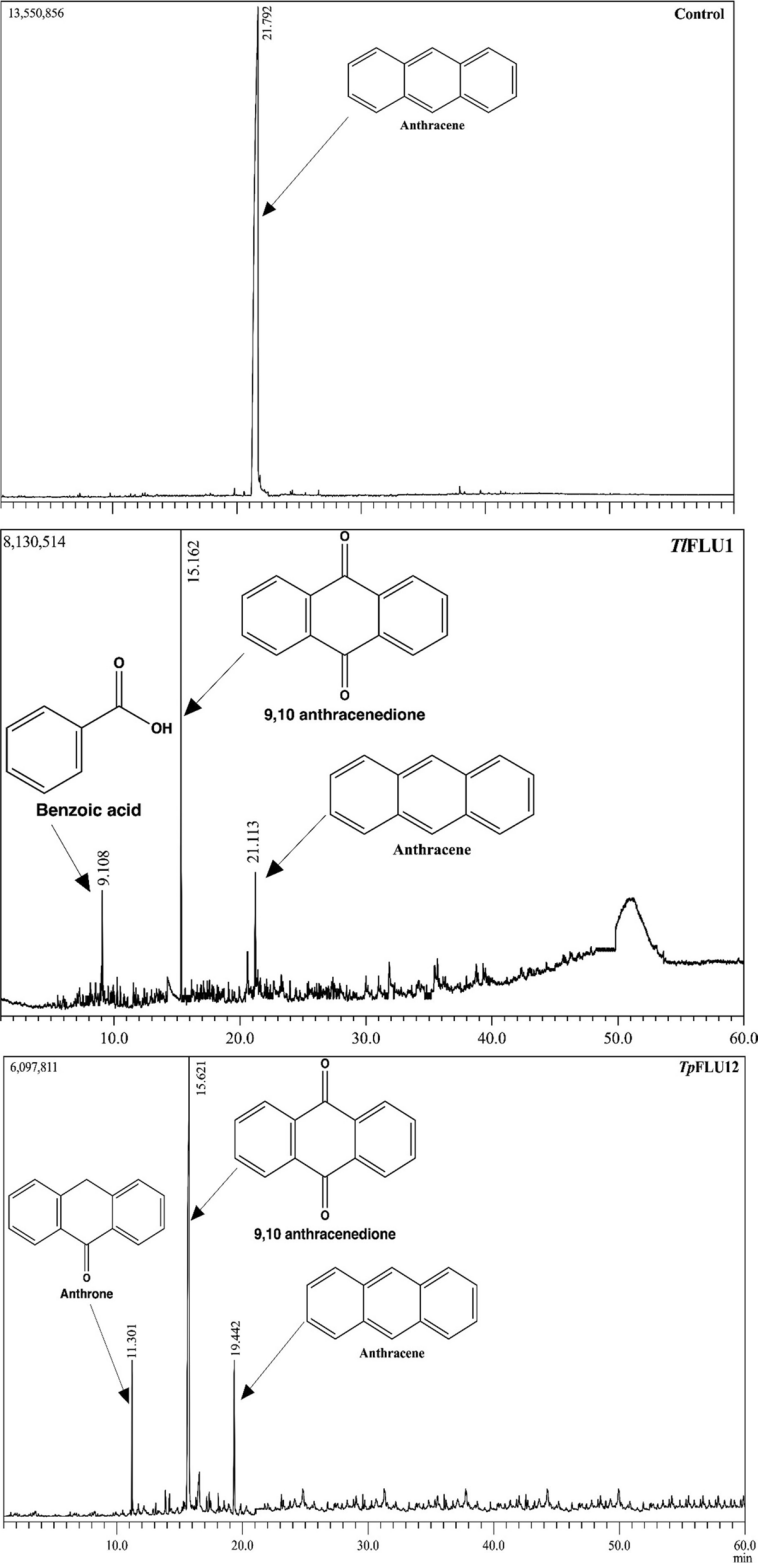
The GC–MS chromatogram of the anthracene mycotransformation peaks identification through the NIST (National Institute of standard technology) library search for control sample (anthracene; 21.792 min), composite sample of *Tl*FLU1 with retention time (9.108 min for Benzoic acid, 15.162 min for 9,10-anthracenedione and 21.113 min for anthracene) and composite sample of *Tp*FLU12 (retention time 11.301 min for anthrone, 15.562 for 9,10-anthracenedione and 19.442 min for anthracene)

**Fig. 4:**
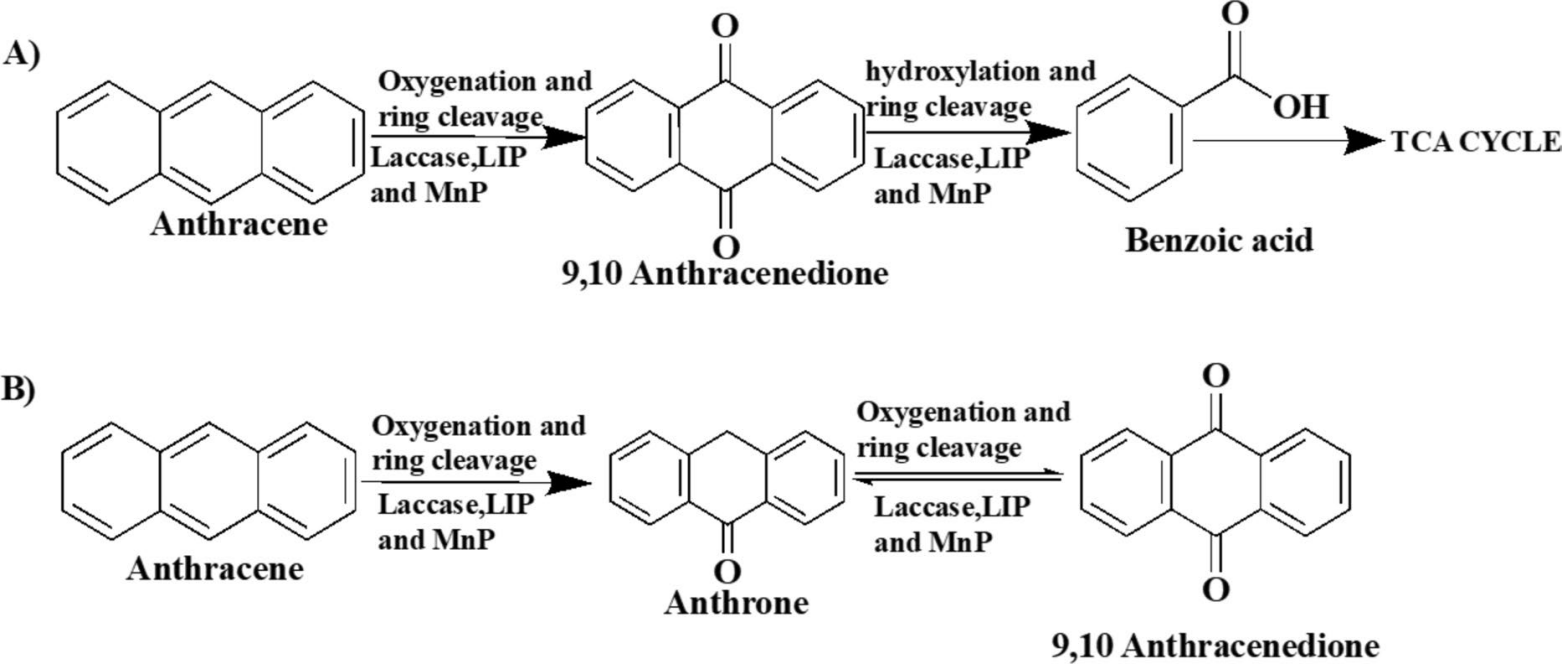
Proposed metabolic pathways for anthracene degradation by *Tl*FLU1(**A**) and *Tp*FLU12 (**B**)

The metabolic products and pathways identified in *Tl*FLU1 and *Tp*FLU12 using GC–MS are shown in Fig. 3, Table 2 and Fig. 4. Two metabolites, 9,10 anthracenedione (molecular ion [M^+^] at m/z 180 and benzoic acid (molecular ion [M^+^] at m/z 105) were observed in the *Tl*FLU1 inoculated flasks with a varying retention time; 15.6 min and 9.1 min, respectively while that of *Tp*FLU12 inoculated flask were anthrone at a retention time of 11.3 min with molecular ion (M^+^) at m/z 195 and 9,10 anthracenedione (RT: 15.2 min) with molecular ion (M^+^) at m/z 180. However, the parent compound (control: anthracene) was detected at a retention time of 21.8 min. Furthermore, the identified metabolic intermediates were used to propose possible anthracene metabolic pathways for each of the fungal strains, as shown in Fig. 4. The theoretical arrangement of the metabolic intermediates suggests that an initial attack at the C-9 and C-10 position of anthracene occurred via oxygenation to form either anthrone or 9,10-anthracenedione before undergoing a spontaneous tautomeric benzoic acid formation as the dead-end product due to hydroxylation and ring cleavage at the C-9 and C-10 position.

**Table 2:** GC–MS profile of the metabolite formed during anthracene degradation

﻿Metabolites	Retention time (min)		Major m/z of fragment ions (% relative abundance)	Tentative identification
	*Tl*FLU1	*Tp*FLU12		
1	21.8		76 (6, M^+^), 88 (4), 89 (8), 150 (4), 151 (6) 152 (7), 176 (14),177 (8), 178 (100), 179 (16)	Anthracene
2	15.6	15.2	50 (19, M^+^), 75 (17), 76 (44), 150 (15), 151 (78), 152 (78), 180 (100), 181 (14), 208 (98), 209 (15)	9,10 anthracenedione
3	11.3	ND	63 (3, M^+^), 82 (7), 139 (4), 163 (7), 164 (6), 165 (53), 166 (10), 193 (9), 194 (100), 195 (15),	Anthrone
4	9.1	ND	50(19, M^+^), 51 (3), 74 (8), 76 (6), 77 (66), 78(6), 105(100), 106 (7),122 (83), 123 (7)	Benzoic acid

*ND* Not detected

### Analysis of the transformed anthracene functional groups

The FTIR spectra of the changes in the functional group of anthracene metabolites observed in the GC–MS analysis are presented in Table 3, Fig. S2. The parent compound (control; anthracene) showed an intense peak at 3020 cm^−1^ and 1275 cm^−1^ due to the C-H stretch and bending of the aromatic ring, while strain *Tl*FLU1 products showed a significant shift in peaks compared to the control set-up. An intense peak was observed at 3170 cm^−1^ due to the C-H vibration stretch of the aromatic rings, followed by O–H stretching of the carboxylic acid at 2800 cm^−1.^ C-H bending of the overtone aromatic ring was recorded at 2000 cm^−1^. An intense peak at 1740 cm^−1^ is linked to the stretching vibration of C=O in the carbonyl and ketone skeletons. Additionally, a phenolic O–H bending was observed at 1275 cm^−1^ coupled with an intense peak at 730 cm^−1^ attributable to the C=C bending of the aromatic ring due to a strong di-substitution.

**Table 3:** Assignment of the infrared spectra absorbance bands of anthracene degradation metabolites

Code	Frequency (cm ^−1^)		Assignment
	*T*lFLU-1	*Tp*FLU-12	
i	3020—3170	3100—3170	C–H vibration stretches of the aromatic ring
ii	2800	ND	O–H stretch in carboxylic acid
iii	2000	ND	C-H bending overtone of the aromatic ring
iv	1740	ND	vibration stretching of C=O in the carbonyl and ketone skeletons
v	1675		C=O stretching of quinone and carboxylic acid dimers
vi	1275		Phenolic O–H bending
vii	1000	ND	C–H bending of the aromatic ring
viii	730		C=C benching of the aromatic ring due to a strong di-substitution

*ND* Not detected

Conversely, in strain *Tp*FLU12 products, a less intense peak was observed compared to strain *Tl*FLU1 products and the parent compound, anthracene. A slight shift to the left with an intense peak at 3100 cm^−1^ was observed due to the C-H vibration on the aromatic rings, followed by C=O stretching of quinone carboxylic acid dimers at 1675 cm^−1^. Furthermore, an intense peak at 1275 cm^−1^ is linked to phenolic O–H bending, while the intense peak at 730 cm^−1^ is attributable to the C=C bending of the aromatic ring due to a strong di-substitution.

### Intracellular and extracellular ligninolytic enzymes profiles during the mycotransformation of anthracene

**Fig. 5:**
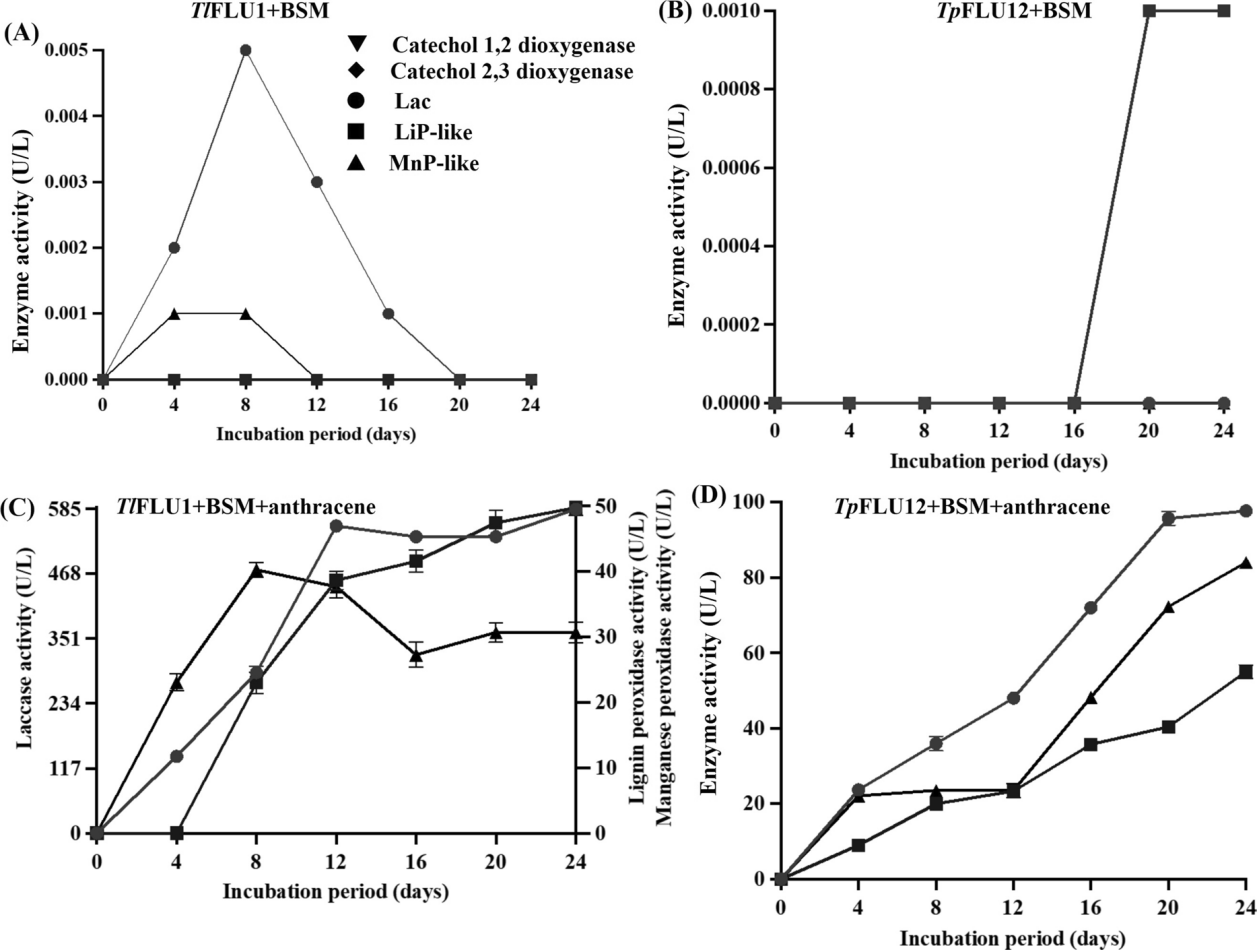
Ligninolytic enzyme activities in response to anthracene degradation by *Tl*FLU1 + BSM **A**, *Tp*FLU12 + BSM **B**, *Tl*FLU1 + BSM + anthracene **C** and *Tp*FLU12 + BSM + anthracene **D**. Curves shown are the activity of Catechol 1,2 dioxygenase (▼), Catechol 2,3 dioxygenase (), Lac (●), LiP-like (■), and MnP-like (▲)

The profiles of intracellular and extracellular ligninolytic enzymes produced by the isolates during the mycotransformation of anthracene are illustrated in Fig. 5. In control experiments, where *Tl*FLU1 was inoculated into flasks containing the culture medium (BSM without anthracene), maximum production of 0.0001 U/L was recorded for catechol 1,2 dioxygenase (C12D) which was constant throughout the incubation period and 0.00013 U/L for catechol 2,3 dioxygenase (C23D) on day 12 while for the ligninolytic enzymes, Lac and MnP-like exhibited a maximum activity of 0.05 U/L and 0.001 U/L, respectively, for on day 8. However, no LiP-like activity was observed throughout the incubation period (Fig. 5a). In contrast, in the *Tp*FLU12 flask, there were no intracellular C12D, C23D, Lac, LiP -like and MnP -like activities observed, except for a brief spike in C12D activity on day 16 with maximum activity of 0.0001 U/L and C23D on day 20 with maximum activity of 0.0001 U/L. In comparison, LiP-like activity reached 0.0010 U/L on day 20 (Fig. 5 b). In *Tl*FLU1 inoculated flasks with anthracene, a substantial increase in extracellular ligninolytic enzyme activity was observed compared to the very low intracellular enzyme activity. Lac, LiP-like and MnP-like reached activity levels of 585 U/L, 49.8 U/L, and 40.3 U/L, respectively, on day 24, with maximum activities achieved on day 12 for Lac and LiP-like and on day 8 for MnP-like while C12D activity reached 7.1 U/L on day 4 and C23D showed a maximum activity of 8.0 U/L on day 8 (Fig. 5c). Similarly, in the *Tp*FLU12 flask, the activities of all ligninolytic enzymes increased steadily over time, culminating at day 24 with maximum activity levels of 97.7 U/L, 55.0 U/L, and 84.1 U/L for Lac, LiP-like, and MnP-like, respectively compared with intracellular enzymes which was so low with maximum activity of 5.3 U/L on day 2 and 3.2 U/L on day 4 for C12D and C23D activities respectively (Fig. 5d).

### Ecotoxicity of culture broth following mycotransformation of anthracene

**Fig. 6:**
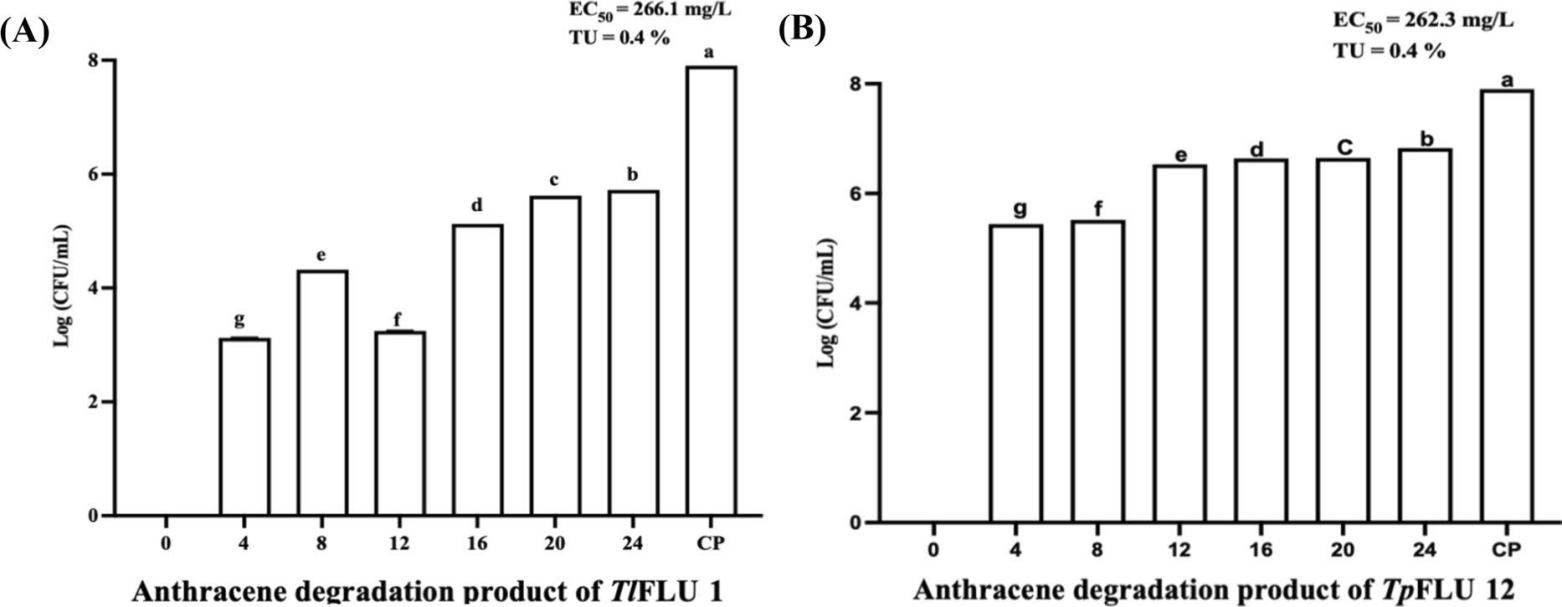
Effective concentration (EC_50_) and toxicity unit (TU) of daily anthracene toxicants to *V. parahaemolyticus (*log CFU/mL) by *Tl*FLU1 **a** and *Tp*FLU12 **b** after 6 h exposure period. Values with different alphabetic superscripts are statistically significant at *p* ≤ 0.05. Key: very toxic (EC_50_ < 1 mg/L); toxic (1 mg/L < EC_50_ ≤ 10 mg/L); harmful (10 mg/L < EC_50_ ≤ 100 mg/L); non-toxic (≥ 100 mg/L), CP (positive control: 400 mg/L anthracene). The data was evaluated using nonlinear regression (log (agonist) vs response model) of colony-forming unit (CFU/mL). Additionally, the bacterium survival data (log CFU/mL) were subjected to analysis of variance (ANOVA) while their means were ranked with Bonferroni’s multiple comparisons test (BMCT) at p ≤ 0.05

The profiles of the surviving population of *V. parahaemolyticus* following exposure to culture extracts collected at different incubation periods are shown in Fig. 6. An increase in *V. parahaemolyticus* survival was observed with an increase in daily anthracene degradation products except for *Tl*FLU1 on day 12 while the maximum survival value of 5.72 Log (CFU/mL) was recorded on day 24. In comparison, a value of 6.82 Log (CFU/mL) was observed on day 24 in *Tp*FLU12 inoculated medium, compared to the positive control (No biotransformation product) with a survival population value of 7.90 Log (CFU/mL) (Fig. 6a). Also, *V. parahaemolyticus* surviving population in all samples collected from *Tl*FLU1 and *Tp*FLU12 inoculated medium is statistically significant (p ≤ 0.05). Effective concentration (EC_50_) and toxicity unit (TU) of 266.1 mg/L and 0.4% were recorded for *Tl*FLU1 inoculated medium, while 262.3 mg/L and TU 0.4% were obtained for *Tp*FLU12 inoculated medium (Fig. 6b).

## Discussion

Fungi, being the most abundant free-living ubiquitous microorganism, are a significant determinant of PAHs fate in both terrestrial and aquatic ecosystems through the mycotransformation process (Ghosal et al. 2016). Mycotransformation of PAHs often commences with high PAH concentration tolerance and low inhibition rate (de Lima Souza et al. 2016). Factors like molecular weight, benzene rings count, concentrations, ﻿pH, and temperature play a major role during PAHs degradation by fungi in the natural environment, which then leads to unprecedented acute and chronic environmental toxicity (Daccò et al. 2020; Lahkar and Deka 2017).

Furthermore, differences in the degradation potential among the isolates may be linked to varying levels of enzyme secretion, enzymatic specificity, or even differences in cell wall permeability and oxidative stress tolerance. For instance, species from the genus *Aspergillus* are known to produce high levels of oxidative enzymes such as laccases and peroxidases, which are critical for PAH breakdown (Aranda 2016; Valiante 2017). The upregulation of key enzymatic pathways observed in this study supports earlier work by Hall et al. (2020), who demonstrated enhanced gene expression of oxidative stress and catabolic enzymes in fungi exposed to aromatic hydrocarbons. These findings underscore the importance of both extracellular and intracellular mechanisms in anthracene degradation and suggest that targeting enzyme induction pathways may further enhance fungal bioremediation strategies.

The indigenous fungal strains *Tl*FLU1 and *Tp*FLU12 used here could utilize anthracene as a carbon and energy source and have been documented as ascomycete strains with a strong affinity for PAHs (Egbewale et al. 2023). However, it is known that complete mineralization of PAHs by fungi starts from their tolerance levels. The data from the solid plating assay (Table S1) revealed that fungi’s tolerance to high anthracene concentrations is not directly proportional to their degradation efficiency but rather a function of ligninolytic enzyme secretion, as shown in Figs. 1 and 4. This observation is consistent with a recent report where tolerance to complex hydrocarbons by fungi was linked to the production of ligninolytic enzymes (Asemoloye et al. 2020). Interestingly, *Tp*FLU12 exhibited a higher anthracene degradation rate (56%) compared to *Tl*FLU1 (38%), despite producing lower biomass (645.6 mg/L vs. 710.3 mg/L, respectively). This phenomenon can be attributed to several metabolic strategies that differ between the two strains. First, *Tp*FLU12 may employ a more efficient extracellular enzyme secretion strategy, as evidenced by the higher specific enzyme activities per unit biomass observed in our enzyme profiling data (Fig. 4). The enhanced secretion efficiency could be related to differences in cell wall permeability, allowing for more effective enzyme export and substrate uptake, as extracellular enzyme activity has been shown to correlate directly with degradation potential across various fungal species (Sack and Günther 1993). Second, the enzymatic specificity of *Tp*FLU12 may be more targeted toward anthracene degradation pathways, utilizing diverse metabolic strategies including cytochrome P450 monooxygenase and ligninolytic pathways that have been demonstrated in other high-efficiency PAH-degrading fungi (Guntupalli et al. 2019), resulting in more efficient substrate transformation. This is supported by the distinct metabolite profiles observed via GC–MS analysis, where *Tp*FLU12 showed more complete conversion to intermediate products and production of less toxic metabolites compared to *Tl*FLU1 (Egbewale et al. 2023). Third, the strain-specific differences in oxidative stress tolerance mechanisms may allow *Tp*FLU12 to maintain higher catalytic efficiency under PAH-induced stress conditions, as suggested by the differential expression patterns of stress-response enzymes observed in our study. Previous studies have shown that differences in ligninolytic enzyme profiles and secretion efficiencies can significantly influence PAH degradation rates among fungal strains (Asemoloye et al. 2018; Bilal et al. 2019). Moreover, the ability to maintain high enzyme activity while producing less biomass suggests that *Tp*FLU12 has evolved more energy-efficient degradation mechanisms, potentially redirecting cellular resources from growth to specialized enzyme production and secretion. This adaptation reflects a strategic allocation of metabolic resources and aligns with the broader principles of microbial energy management, where organisms balance growth and maintenance costs through optimized metabolic pathways (McKinlay et al. 2020). Dynamic flux balance modeling supports this view, showing that extracellular enzyme production, although energetically expensive, is essential for degrading complex substrates and can be modulated based on environmental conditions (Quintin et al. 2021). Furthermore, the competitive dynamic enzyme allocation (CODEAL) model indicates that enzyme allocation strategies, shaped by substrate saturation and competitive dynamics, can maximize metabolic efficiency while minimizing cost (Qi et al. 2023). Thus, *Tp*FLU12’s metabolic profile may represent a fine-tuned system optimized for anthracene degradation through high enzyme specificity and secretion efficiency, consistent with energy-conserving strategies observed in indigenous fungal strains adapted for PAH bioremediation (Egbewale et al. 2023, 2024). Further enzyme profiling and transcriptomic analysis could elucidate the underlying metabolic strategies responsible for this disparity. Similarly, other studies have reported high degradation rates of anthracene by various fungal strains, such as *Aspergillus fumigatus* (60% after 5 days) (Ye et al. 2011), *Pycnoporus sanguineus* H1 (67.5% under in vivo conditions after 14 days) (Zhang et al. 2015), and *Armillaria* sp. F022 (92% within 30 days) (Hadibarata et al. 2013). Moreover, ﻿pH was observed to play a significant role during anthracene mycotransformation with a slight reduction in the pH medium of *Tl*FLU1 inoculated flask (Fig. 2). This observation could be attributed to the production of some acidic metabolites, such as benzoic acid during the degradation process (Ye et al. 2011), Similarly, a recent study (Vipotnik et al. 2021), demonstrates the ability of ascomycetes fungi to optimally degrade PAHs such as fluorene, pyrene, BaP effectively at a pH of 5 and 7 which corroborate the results from the present study. Additionally, the increased media pH recorded in *Tp*FLU12 could be ascribed to anthracene metabolism linked to producing a ligninolytic enzyme (Aranda et al. 2017). The complete metabolism of a PAH compound at a pH close to the current study (pH 7) due to the induction of ligninolytic enzymes during the optimization process is also documented (Bankole et al. 2021). Also, a previous study revealed that fungi’s metabolism of polyaromatic compounds is best enhanced at a pH range between 7 and 9 (Batista-garcía et al. 2017).

The degradation kinetics is a highly imperative tool for assessing total pollutant removal (Ortega-González et al. 2015). The observed zero and first-order kinetics fitting during the anthracene degradation by these indigenous fungi attest to the general proposition for microbial metabolism of PAHs, especially fungi (Jauhari et al. 2020; Mishra et al. 2014). The low rate constant (*K*) and high half-life (t_1/2_) recorded in the first-order kinetics indicate that anthracene degradation by these fungi is both concentration and time-dependent (Mandal et al. 2018a).

The GC–MS analysis established that anthracene mycotransformation proceeds through an initial attack at the C-9 and C-10 positions of anthracene via oxygenation to form either anthrone or 9,10-anthracenedione before undergoing a spontaneous tautomeric benzoic acid formation due to the hydroxylation and ring cleavage at the C-9 and C-10 positions (Table 2). This novel metabolic pathway has been identified during anthracene degradation by the marine fungi *Cladosporium sp*. CBMAI 1237 (Birolli et al. 2018). A similar observation (Jove et al. 2016), where anthracene degradation pathways in fungi were initiated at C-9 and C-10 to form anthraquinone and end with the formation of phthalic acid, is available. However, the appearance of benzoic acid in the pathway described in the present study (Fig. 3) is not uncommon because phthalic acid and benzoic acid are known as a gateway to the TCA cycle during anthracene metabolism (Hadibarata et al. 2012). Also, the appearance of a quinone derivative (9,10-anthracenedione) as a dead-end product during anthracene degradation by isolate *Tp*FLU12 is not unusual in the anthracene metabolic pathway (Aranda et al. 2017).

The FTIR ﻿spectrum results (Table 3, Fig. S2) further strengthen the proposed anthracene degradation pathways, with products consisting of quinones derivatives, C-H stretch and bending of the aromatic ring, stretching O–H in carboxylic acid to form benzoic acid, expansion of C=O in carboxylic acids, O–H stretching of the carboxylic acid, C=O in carbonyl and ketones skeleton, bending of phenol O–H, C–C expansion in aromatic rings, C-H vibration of the aromatic rings and C=C–H: C–H bend in alkanes. The appearance of these functional groups has been attributed to features of the PAHs metabolic pathway during the degradation of pyrene by the *C. byrsina* strain APC5 (Agrawal and Shahi 2017). A similar functional group has been documented while yeast-mediated degradation of BaP using a 3-level Box-Behnken design (Mandal et al. 2018b). Also, the presence of the O–H dimer is indicative of the complete degradation of anthracene into less toxic products (Arunprasath et al. 2019).

Recent studies have confirmed that fungi specifically from the ascomycota phylum often employ a variety of enzymes, including LiP, versatile peroxidase, MnP, Lac, cytochrome P450 monooxygenases, and epoxide hydrolases to degrade PAHs (Aranda 2016; Balaji et al. 2014; Kadri et al. 2017; Pozdnyakova 2012). This study observed that the interplay between intracellular (C12D and C23D) and extracellular enzymes (Lac, LiP-like, and MnP-like) produced by ascomycete fungi (*Tl*FLU1 and *Tp*FLU12) plays a crucial role in the mycotransformation process of anthracene. This observation is consistent with previous studies, which linked these enzymes to the mechanism used by yeast consortia during the breakdown of perylene and benzo[ghi]perylene individually when used as a sole carbon source (Mandal and Das 2018). Similar enzymes have been reported in *Trichoderma* sp. 109, a strain akin to *Tl*FLU1 in phenanthrene degradation as a sole carbon source (Hadibarata et al. 2007). The role of intracellular enzymes C12D and C23D are of significant interest because they seem to be produced at their highest levels in the whole fungi at different stages (4 and 8-d respectively). This suggests they might be the first step in how anthracene is transformed. However, it was not entirely clear how involved these intracellular enzymes were because they were found in much lower amounts compared to the extracellular ligninolytic enzymes (Lac, LiP-like, and MnP-like) in both the whole fungi and the broth, regardless of whether anthracene was present. The most probable explanation for detecting these intracellular enzymes in the early stage of the transformation process could be linked to the fungal strain’s defense system against oxidative stress, reactive oxygen species, and ATP synthesis. This opinion aligns with a previous report (Zafra et al. 2015) where MnP-like peroxidase activity was detected during the early stage of PAHs degradation, which was linked to response against oxidative stress rather than being involved in the degradation of PAHs by *T. asperellum* H15. Therefore, the role of intracellular enzymes C12D and C23D in fungi, particularly in the early stages of anthracene transformation, is likely linked to the fungal strain’s defense system against oxidative stress and ATP synthesis (Camacho-Morales et al. 2018; Lebeda et al. 2001). These enzymes are part of the fungal cell wall integrity signaling pathway, which produces secondary metabolites (Valiante 2017). Additionally, the structural analysis of anthracene derivatives like anthraquinone-fused enediynes sheds light on their biosynthetic pathways and their potential defense system mechanisms, interactions with reactive oxygen species due to the production of intracellular enzymes like C12D and C23 (Hall et al. 2020; Kharlamova 2022). However, another potential intracellular enzyme, cytochrome P450, might be responsible for the initial transformation mechanism used by these fungi since they have been reported in *Penicillium oxalicum* when anthracene was used as a carbon source during its transformation process (Aranda et al. 2017). Further, overexpression of lac, LiP-like, and MnP-like genes was observed in PAH-contaminated marine sediments belonging to Eurotiales, specifically *Talaromyces helices* and *Trichoderma* sp. during PAHs biotransformation (Álvarez-Barragán et al. 2021). An account of the induction of Lac, LP-like, and MnP-like during the removal of manganese and phenanthrene with a removal efficiency of 92.17 and 93.85%, respectively, by *P. eryngii* is also reported (Wu et al. 2016). Similarly, some authors (Teerapatsakul et al. 2016) implicated the secretion of LP-like, ﻿MnP-like, and Lac by T. polyzona RYNF13 in the degradation of fluorene, ﻿phenanthrene, and pyrene. In addition, LiP-like, MnP-like, and Lac have been linked to ring cleavage via oxidation, carboxylation, and decarboxylation in various PAHs degradation pathways (Bankole et al. 2021). The significant role played by ligninolytic enzymes in the degradation of complex hydrocarbons, such as aliphatic and PAH, by the ascomycete fungus, *Penicillium* sp. CHY-2 was demonstrated for the degradation of decane (49.0%), butylbenzene (42.0%) and dodecane (33.0%) and naphthalene (15.0%), acenaphthene (10.0%), octane (8.0%), ethylbenzene (4.0%) and benzo[a]pyrene (2.0%) (Govarthanan et al. 2017).

Studies on PAHs’ mycotransformation should not be limited to their complete disappearance alone or transient product formation, but the assessment of toxic intermediate products formed to evaluate their actual environmental impact. The ecotoxicity assay data revealed that metabolites formed induced acute toxicity despite a significant reduction in parent compound concentration. A similar report (Mtibaà et al. 2018b) is also available, where metabolites of bisphenol degradation showed higher toxicity than bisphenol. The low survival of marine bacteria exposed to toxicants (degradation products), in contrast to the high survival in the positive control group (No PAHs), could be attributed to the formation of oxygen-containing PAHs, specifically quinone derivatives. Quinone derivatives are recognized for their role as direct-acting mutagens, as noted in previous studies (Chibwe et al. 2017; Menzie et al. 1992), further underscoring their capacity to induce toxicity. Although an increase in the surviving population of the marine bacterium after exposure to the anthracene-containing medium undergoing myco-degradation demonstrated a reduction in acute toxicity of the parent compound over treatment time, the observed reduced population on day 12 of *Tl*FLU1 inoculated medium (Fig. 5) revealed that toxicity level is not a function of contaminant concentration (Lukić et al. 2016). In addition, the recorded high EC_50_ and low TU values indicate that metabolic products accumulate during the mycotransformation process as a non-toxic product (Arunprasath et al. 2019). The observed reduction in the population of the marine bacterium on day 12, toxicant from *Tl*FLU1 could be attributed to the toxicity due to aromatic structure with *ortho-substituted* C=O and –OH groups formed during the degradation process (Kamaya et al. 2005). Also, the observed reduction in toxicity suggests that marine bacteria can be used as a cheap bioassay protocol in the environmental risk assessment of organo-pollutants like anthracene and its metabolites because of their sensitivity to toxicants (Strotmann et al. 2020).

## Conclusions

The study ascertained the tolerance levels of the fungal strains to varying anthracene concentrations on a solid agar plate, the role of ligninolytic enzymes in response to anthracene tolerance and its mycotransformation efficiency, kinetics, and the detection of intermediates formed along with the effects on *Vibrio parahaemolyticus* survival. Both isolates under study, *Tl*FLU1 *and Tp*FLU12, could tolerate anthracene concentration up to 1000 mg/L, with ligninolytic enzymes linked to the tolerance rate and degradation efficiency. Intracellular enzymes could be involved in the initial mechanism employed by these fungi for the transformation process or may have been produced as a defense system against oxidative stress, reactive oxygen species, and ATP synthesis. However, further genomics and secretome studies are required to confirm this observation. Both isolates best fit zero and first-order kinetics, leading to the accumulation of non-toxic transient products. The high EC_50_ and low TU values suggest that the accumulated toxicants in the anthracene degradation pathway are toxic. Additionally, the presence of aromatic structures featuring *ortho-substituted* C=O and –OH groups was linked to a noteworthy decrease in the survival of marine bacteria when exposed to anthracene mycotransformation toxicants compared to the positive control (no anthracene or mycotransformation products present). Thus, this observation highlights the possible use of these fungal strains in the amelioration of PAH-contaminated sites and supports the notion of using solid plate techniques as a cheap, fast, and sustainable procedure in selecting a high PAH tolerance strain that is effective in degrading PAHs to non-toxic by-products.

## Supplementary Information

Below is the link to the electronic supplementary material.

Supplementary file1 (PDF 392 KB)

## Data Availability

Data are contained within the article or the Supplementary Material.

## References

[CR1] Adenipekun CO, Ipeaiyeda AR, Olayonwa AJ, Egbewale SO (2015) Biodegradation of polycyclic aromatic hydrocarbons (PAHs) in spent and fresh cutting fluids contaminated soils by Pleurotus pulmonarius (Fries). Quelet and Pleurotus ostreatus (Jacq.) Fr. P. Kumm. Afr J Biotechnol 14:661–667. 10.5897/AJB2014.14187

[CR2] Adnan LA, Sathishkumar P, Yusoff ARM, Hadibarata T, Ameen F, Rahim A, Yusoff M (2016) Rapid bioremediation of Alizarin Red S and Quinizarine Green SS dyes using *Trichoderma lixii* F21 mediated by biosorption and enzymatic processes. Bioprocess Biosyst Eng 40:85–97. 10.1007/s00449-016-1677-7 27663440 10.1007/s00449-016-1677-7

[CR3] Agrawal N, Shahi SK (2017) Degradation of polycyclic aromatic hydrocarbon (pyrene) using novel fungal strain *Coriolopsis byrsina* strain APC5. Int Biodeterior Biodegrad 122:69–81. 10.1016/j.ibiod.2017.04.024

[CR4] Álvarez-Barragán J, Cravo-Laureau C, Wick LY, Duran R (2021) Fungi in PAH-contaminated marine sediments: cultivable diversity and tolerance capacity towards PAH. Mar Pollut Bull 164:112082. 10.1016/j.marpolbul.2021.112082 33524832 10.1016/j.marpolbul.2021.112082

[CR5] Aranda E (2016) Promising approaches towards biotransformation of polycyclic aromatic hydrocarbons with Ascomycota fungi. Curr Opin Biotechnol 38:1–8. 10.1016/j.copbio.2015.12.002 26722717 10.1016/j.copbio.2015.12.002

[CR6] Aranda E, Godoy P, Reina R, Badia-Fabregat M, Rosell M, Marco-Urrea E, García-Romera I (2017) Isolation of Ascomycota fungi with capability to transform PAHs: insights into the biodegradation mechanisms of *Penicillium oxalicum*. Int Biodeterior Biodegrad 122:141–150. 10.1016/j.ibiod.2017.05.015

[CR7] Argumedo-Delira R, Alarcón A, Ferrera-Cerrato R, Almaraz JJ, Peña-Cabriales JJ (2012) Tolerance and growth of 11 Trichoderma strains to crude oil, naphthalene, phenanthrene and benzo[a]pyrene. J Environ Manage 95:S291–S299. 10.1016/j.jenvman.2010.08.011 20869805 10.1016/j.jenvman.2010.08.011

[CR8] Arunprasath T, Sudalai S, Meenatchi R, Jeyavishnu K, Arumugam A (2019) Biodegradation of triphenylmethane dye malachite green by a newly isolated fungus strain. Biocatal Agric Biotechnol 17:672–679. 10.1016/j.bcab.2019.01.030

[CR9] Asemoloye MD, Ahmad R, Jonathan SG (2018) Transcriptomic responses of catalase, peroxidase and laccase encoding genes and enzymatic activities of oil spill inhabiting rhizospheric fungal strains. Environ Pollut 235:55–64. 10.1016/j.envpol.2017.12.042 29274538 10.1016/j.envpol.2017.12.042

[CR10] Asemoloye MD, Tosi S, Daccò C, Wang X, Xu S, Marchisio MA, Gao W, Jonathan SG, Pecoraro L (2020) Hydrocarbon degradation and enzyme activities of *Aspergillus oryzae* and *Mucor irregularis* isolated from nigerian crude oil-polluted sites. Microorganisms 8:1–19. 10.3390/microorganisms8121912 10.3390/microorganisms8121912 PMC7761101 33266344

[CR11] Balaji V, Arulazhagan P, Ebenezer P (2014) Enzymatic bioremediation of polyaromatic hydrocarbons by fungal consortia enriched from petroleum contaminated soil and oil seeds. J Environ Biol 35:521–529 24813008

[CR12] Bankole PO, Semple KT, Jeon BH, Govindwar SP (2021) Biodegradation of fluorene by the newly isolated marine-derived fungus, *Mucor irregularis* strain bpo1 using response surface methodology. Ecotoxicol Environ Saf 208:111619–111619. 10.1016/j.ecoenv.2020.111619 33396139 10.1016/j.ecoenv.2020.111619

[CR13] Baran A, Tarnawski M, Urbański K, Klimkowicz-Pawlas A, Spałek I (2017) Concentration, sources and risk assessment of PAHs in bottom sediments. Environ Sci Pollut Res 24:23180–23195. 10.1007/s11356-017-9944-y 10.1007/s11356-017-9944-y PMC5630654 28828716

[CR14] Batista-garcía A, Vinoth V, Ariste A, Tovar-herrera OE, Savary O, Peidro-guzm H, Jackson SA, Dobson ADW, Gonz D, Folch-mallol JL, Leduc R, Rayo S, Cabana H (2017) Simple screening protocol for identification of potential mycoremediation tools for the elimination of polycyclic aromatic hydrocarbons and phenols from hyperalkalophile industrial effluents. J Environ Manage 198:1–11. 10.1016/j.jenvman.2017.05.010 28499155 10.1016/j.jenvman.2017.05.010

[CR15] Bilal M, Adeel M, Rasheed T, Zhao Y, Iqbal HMN (2019) Emerging contaminants of high concern and their enzyme-assisted biodegradation – a review. Environ Int 124:336–353. 10.1016/j.envint.2019.01.011 30660847 10.1016/j.envint.2019.01.011

[CR16] Birolli WG, de A. Santos D, Alvarenga N, Garcia ACFS, Romão LPC, Porto ALM (2018): Biodegradation of anthracene and several PAHs by the marine-derived fungus *Cladosporium* sp. CBMAI 1237. *Mar Pollut Bull* 129, 525–533. 10.1016/j.marpolbul.2017.10.023 10.1016/j.marpolbul.2017.10.023 29055563

[CR17] Camacho-Morales RL, García-Fontana C, Fernández-Irigoyen J, Santamaría E, González-López J, Manzanera M, Aranda E (2018) Anthracene drives sub-cellular proteome-wide alterations in the degradative system of *Penicillium oxalicum*. Ecotoxicol Environ Saf 159:127–135. 10.1016/j.ecoenv.2018.04.051 29734068 10.1016/j.ecoenv.2018.04.051

[CR18] Chibwe L, Davie-Martin CL, Aitken MD, Hoh E, Massey Simonich SL (2017) Identification of polar transformation products and high molecular weight polycyclic aromatic hydrocarbons (PAHs) in contaminated soil following bioremediation. Sci Total Environ 599:1099–1107. 10.1016/j.scitotenv.2017.04.190 28511355 10.1016/j.scitotenv.2017.04.190

[CR19] Daccò C, Nicola L, Temporiti MEE, Mannucci B, Corana F, Carpani G, Tosi S (2020) Trichoderma: evaluation of its degrading abilities for the bioremediation of hydrocarbon complex mixtures. Appl Sci 10:3152–3152. 10.3390/APP10093152

[CR20] de Lima Souza HM, Sette LDe, da Mota AJ, do Nascimento Neto JF, Rodrigues A, de Oliveira TsB, de Oliveira FM, de Oliveira LAn, dos Santos Barroso H, Zanotto SP (2016): Filamentous fungi isolates of contaminated sediment in the amazon region with the potential for benzo(a)pyrene degradation. *Water Air Soil Pollut* 227, 431. 10.1007/s11270-016-3101-y

[CR21] Eeshwarasinghe D, Loganathan P, Vigneswaran S (2019) Simultaneous removal of polycyclic aromatic hydrocarbons and heavy metals from water using granular activated carbon. Chemosphere 223:616–627. 10.1016/j.chemosphere.2019.02.033 30798057 10.1016/j.chemosphere.2019.02.033

[CR22] Egbewale SO, Kumar A, Mokoena MP, Olaniran AO (2023) Metabolic biodegradation pathway of fluoranthene by indigenous *Trichoderma lixii* and *Talaromyces pinophilus* spp. Catalysts. 10.3390/catal13050791

[CR23] Egbewale SO, Kumar A, Olasehinde TA, Mokoena MP, Olaniran AO (2024) Anthracene detoxification by Laccases from indigenous fungal strains *Trichoderma lixii* FLU1 and *Talaromyces pinophilus* FLU12. Biodegradation 35:769–787. 10.1007/s10532-024-10084-3 38822999 10.1007/s10532-024-10084-3 PMC11246312

[CR24] Ellickson KM, Herbrandson C, Krause MJ, Pratt GC, Kellock KA (2020) Comparative risk estimates of an expanded list of PAHs from community and source-influenced air sampling. Chemosphere 253:126680–126680. 10.1016/j.chemosphere.2020.126680 32289604 10.1016/j.chemosphere.2020.126680

[CR25] Gao JP, Maguhn J, Spitzauer P, Kettrup A (1998) Distribution of polycyclic aromatic hydrocarbons (PAHs) in pore water and sediment of a small aquatic ecosystem. Int J Environ Anal Chem 69:227–242. 10.1080/03067319808032589

[CR26] Ghosal D, Ghosh S, Dutta TK, Ahn Y (2016) Current state of knowledge in microbial degradation of polycyclic aromatic hydrocarbons (PAHs): a review. Front Microbiol 7:1369. 10.3389/fmicb.2016.01369 27630626 10.3389/fmicb.2016.01369 PMC5006600

[CR27] Govarthanan M, Fuzisawa S, Hosogai T, Chang Y-C (2017) Biodegradation of aliphatic and aromatic hydrocarbons using the filamentous fungus *Penicillium* sp. CHY-2 and characterization of its Manganese peroxidase activity. RSC Adv 7:20716–20723. 10.1039/C6RA28687A

[CR28] Guntupalli S, Thunuguntla V, Chalasani LM, Rao CV, Bondili JS (2019) Degradation and metabolite profiling of benz (a) anthracene, dibenz (a, h) anthracene and indeno [1, 2, 3-cd] pyrene by *Aspergillus terricola*. Polycycl Aromat Comp 39:84–92. 10.1080/10406638.2016.1262878

[CR31] Hadibarata T, Kristanti RA (2012) Identification of metabolites from benzo[a]pyrene oxidation by ligninolytic enzymes of *Polyporus* sp. S133. J Environ Manage 111:115–119. 10.1016/j.jenvman.2012.06.044 22835655 10.1016/j.jenvman.2012.06.044

[CR29] Hadibarata T, Tachibana S, Itoh K (2007) Biodegradation of phenanthrene by fungi screened from nature. Pak J Biol Sci 10:2535–2543. 10.3923/pjbs.2007.2535.2543 19070127 10.3923/pjbs.2007.2535.2543

[CR30] Hadibarata T, Khudhair AB, Salim MR (2012) Breakdown products in the metabolic pathway of anthracene degradation by a ligninolytic fungus *Polyporus* sp. S133. Water Air Soil Pollut 223:2201–2208. 10.1007/s11270-011-1016-1

[CR32] Hadibarata T, Zubir MMFA, Rubiyatno CTZ, Yusoff ARM, Salim MR, Fulazzaky MA, Seng B, Nugroho AE (2013) Degradation and transformation of anthracene by white-rot fungus *Armillaria* sp. F022. Folia Microbiol 58:385–391. 10.1007/s12223-013-0221-2 23307571 10.1007/s12223-013-0221-2

[CR33] Hall KR, Robins KJ, Williams EM, Rich MH, Calcott MJ, Copp JN, Little RF, Schwörer R, Evans GB, Patrick WM (2020) Intracellular complexities of acquiring a new enzymatic function revealed by mass-randomisation of active-site residues. Elife 9:e59081. 10.7554/eLife.59081 33185191 10.7554/eLife.59081 PMC7738182

[CR34] Hammel KE, Green B, Gai WZ (1991) Ring fission of anthracene by a eukaryote. PNAS USA 88:10605–10608. 10.1073/pnas.88.23.106 1961727 10.1073/pnas.88.23.10605 PMC52978

[CR35] Jajoo A, Mekala NR, Tomar RS, Grieco M, Tikkanen M, Aro EM (2014) Inhibitory effects of polycyclic aromatic hydrocarbons (PAHs) on photosynthetic performance are not related to their aromaticity. J Photochem Photobiol B Biol 137:151–155. 10.1016/j.jphotobiol.2014.03.011 10.1016/j.jphotobiol.2014.03.011 24793324

[CR36] Jarque S, Masner P, Klánová J, Prokeš R, Bláha L (2016) Bioluminescent *Vibrio fischeri* assays in the assessment of seasonal and spatial patterns in toxicity of contaminated river sediments. Front Microbiol 7:1–11. 10.3389/fmicb.2016.01738 27872614 10.3389/fmicb.2016.01738 PMC5097916

[CR37] Jauhari N, Mishra S, Kumari B, Singh SN, Chauhan PS, Upreti DK (2020) Bacteria induced degradation of anthracene mediated by catabolic enzymes. Polycycl Aromat Compd 40:313–325. 10.1080/10406638.2017.1420667

[CR38] Jove P, Olivella MA, Camarero S, Caixach J, Planas C, Cano L, De Las Heras FX (2016) Fungal biodegradation of anthracene-polluted cork: A comparative study. *J Environ Sci Health - Toxic/Hazard Subst*. Environ Eng 51:70–77. 10.1080/10934529.2015.1079114 10.1080/10934529.2015.1079114 26540209

[CR39] Kadri T, Rouissi T, Brar SK, Cledon M, Sarma S, Verma M (2017) Biodegradation of polycyclic aromatic hydrocarbons (PAHs) by fungal enzymes: a review. J Environ Sci 51:52–74. 10.1016/j.jes.2016.08.023 10.1016/j.jes.2016.08.023 28115152

[CR40] Kamaya Y, Fukaya Y, Suzuki K (2005) Acute toxicity of benzoic acids to the crustacean *Daphnia magna*. Chemosphere 59:255–261. 10.1016/j.chemosphere.2004.11.003 15722097 10.1016/j.chemosphere.2004.11.003

[CR41] Kharlamova T (2022): Anti-fungal activity of anthraquinone derivatives. *Chem J Kazakhstan* 2, 26–47. 10.51580/2022-2/2710-1185.63

[CR42] Kimani V, Ullrich R, Büttner E, Herzog R, Kellner H, Jehmlich N, Hofrichter M, Liers C (2021) First dye-decolorizing peroxidase from an ascomycetous fungus secreted by *Xylaria grammica*. Biomolecules 11:1391 34572604 10.3390/biom11091391 PMC8469222

[CR43] Lahkar J, Deka H (2017) Isolation of polycyclic aromatic hydrocarbons (PAHs) degrading fungal candidate from oil-contaminated soil and degradation potentiality study on anthracene. Polycycl Aromat Compd 37:141–147. 10.1080/10406638.2016.1220957

[CR44] Lebeda A, Luhová L, Sedlářová M, Jančová D (2001): The role of enzymes in plant-fungal pathogens interactions. *J Plant Diseases Protect*, 89–111. https://www.jstor.org/stable/43215387

[CR45] Li X, Zhang S, Guo R, Xiao X, Liu B, Mahmoud RK, Abukhadra MR, Qu R, Wang Z (2024) Transformation and degradation of pah mixture in contaminated sites: Clarifying their interactions with native soil organisms. Toxics 12:361. 10.3390/toxics12050361 38787140 10.3390/toxics12050361 PMC11126024

[CR46] Lin Z, Du J, Yin K, Wang L, Yu H (2004) Mechanism of concentration addition toxicity: they are different for nonpolar narcotic chemicals, polar narcotic chemicals and reactive chemicals. Chemosphere 54:1691–1701. 10.1016/J.CHEMOSPHERE.2003.09.031 14675847 10.1016/j.chemosphere.2003.09.031

[CR47] Lukić B, Panico A, Huguenot D, Fabbricino M, van Hullebusch ED, Esposito G (2016) Evaluation of PAH removal efficiency in an artificial soil amended with different types of organic wastes. Euro-Mediterr J Environ Integr. 10.1007/s41207-016-0001-x

[CR48] Mandal SK, Das N (2018) Biodegradation of perylene and benzo [ghi] perylene (5–6 rings) using yeast consortium: kinetic study, enzyme analysis and degradation pathway. J Environ Biol 39:5–15. 10.22438/jeb/39/1/MRN-540

[CR49] Mandal SK, Ojha N, Das N (2018a) Process optimization of benzo [ghi ] perylene biodegradation by yeast consortium in presence of ZnO nanoparticles and produced biosurfactant using Box-Behnken design. Front Biol. 10.1007/s11515-018-1523-1

[CR50] Mandal SK, Ojha N, Das N (2018b) Optimization of process parameters for the yeast mediated degradation of benzo[a]pyrene in presence of ZnO nanoparticles and produced biosurfactant using 3-level Box-Behnken design. Ecol Eng 120:497–503. 10.1016/j.ecoleng.2018.07.006

[CR51] McKinlay JB, Cook GM, Hards K (2020): Microbial energy management—a product of three broad tradeoffs, *Advances in Microbial Physiology*. Elsevier, pp. 139–185. 10.1016/bs.ampbs.2020.09.001 10.1016/bs.ampbs.2020.09.001 34756210

[CR52] Menzie CA, Potocki BB, Santodonato J (1992) Exposure to carcinogenic PAHs in the environment. Environ Sci Technol 26:1278–1284. 10.1021/es00031a002

[CR53] Mishra S, Singh SN, Pande V (2014) Bacteria induced degradation of fluoranthene in minimal salt medium mediated by catabolic enzymes in vitro condition. Bioresour Technol 164:299–308. 10.1016/j.biortech.2014.04.076 24862007 10.1016/j.biortech.2014.04.076

[CR54] Morgenstern I, Klopman S, Hibbett DS (2008) Molecular evolution and diversity of lignin degrading heme peroxidases in the Agaricomycetes. J Mol Evol 66:243–257. 10.1007/s00239-008-9079-3 18292958 10.1007/s00239-008-9079-3

[CR55] Mtibaà R, Barriuso J, de Eugenio L, Aranda E, Belbahri L, Nasri M, Martínez MJ, Mechichi T (2018a) Purification and characterization of a fungal laccase from the ascomycete *Thielavia* sp. and its role in the decolorization of a recalcitrant dye. Int Biodeterior Biodegrad 120:1744–1751. 10.1016/j.ijbiomac.2018.09.175 10.1016/j.ijbiomac.2018.09.175 30268749

[CR56] Mtibaà R, Olicón-Hernández DR, Pozo C, Nasri M, Mechichi T, González J, Aranda E (2018b) Degradation of bisphenol A and acute toxicity reduction by different thermo-tolerant ascomycete strains isolated from arid soils. Ecotoxicol Environ Saf 156:87–96. 10.1016/j.ecoenv.2018.02.077 29533211 10.1016/j.ecoenv.2018.02.077

[CR57] Nacher-Mestre J, Serrano R, Portoles T, Berntssen MHG, Perez-Sanchez J, Hernandez F (2014) Screening of pesticides and polycyclic aromatic hydrocarbons in feeds and fish tissues by gas chromatography coupled to high-resolution mass spectrometry using atmospheric pressure chemical ionization. J Agric Food Chem 62:2165–2174. 10.1021/jf405366n 24559176 10.1021/jf405366n

[CR58] Ortega-González DK, Cristiani-Urbina E, Flores-Ortíz CM, Cruz-Maya JA, Cancino-Díaz JC, Jan-Roblero J (2015) Evaluation of the removal of pyrene and fluoranthene by *Ochrobactrum anthropi*, *Fusarium* sp. and their coculture. Appl Biochem Biotechnol 175:1123–1138. 10.1007/s12010-014-1336-x 25369894 10.1007/s12010-014-1336-x

[CR59] Patel H, Gupte A, Gupte S (2009) Biodegradation of fluoranthene by basidiomycetes fungal isolate *Pleurotus ostreatus* HP-1. Appl Biochem Biotechnol 157:367–376. 10.1007/s12010-008-8286-0 18574565 10.1007/s12010-008-8286-0

[CR60] Pozdnyakova NN (2012) Involvement of the ligninolytic system of White-Rot and litter-decomposing fungi in the degradation of polycyclic aromatic hydrocarbons. Biotechnol Res Int 2012:20–20. 10.1155/2012/243217 10.1155/2012/243217 PMC3398574 22830035

[CR61] Qi S, Wang G, Li W, Zhou S (2023) Exploring the competitive dynamic enzyme allocation scheme through enzyme cost minimization. ISME Commun 3:121. 10.1038/s43705-023-00331-8 37985704 10.1038/s43705-023-00331-8 PMC10662282

[CR62] Quintin M, Dukovski I, Bhatnagar J, Segrè D (2021): Optimality of extracellular enzyme production and activity in dynamic flux balance modeling. *bioRxiv*, 2021.11.01.466736. 10.1101/2021.11.01.466736

[CR63] Rengarajan T, Rajendran P, Nandakumar N, Lokeshkumar B, Rajendran P, Nishigaki I (2015) Exposure to polycyclic aromatic hydrocarbons with special focus on cancer. Asian Pac J Trop Biomed 5:182–189. 10.1016/S2221-1691(15)30003-4

[CR64] Rotini A, Manfra L, Spanu F, Pisapia M, Cicero AM, Migliore L (2017): Ecotoxicological method with marine bacteria *Vibrio anguillarum* to evaluate the acute toxicity of environmental contaminants. *J Vis Exp*, 55211–55211. 10.3791/55211 10.3791/55211 PMC5608138 28605381

[CR65] Sack U, Günther T (1993) Metabolism of PAH by fungi and correlation with extracellular enzymatic activities. J Basic Microbiol 33:269–277. 10.1002/jobm.3620330411 8229670 10.1002/jobm.3620330411

[CR66] Saraswathy A, Hallberg R (2002) Degradation of pyrene by indigenous fungi from a former gasworks site. FEMS Microbiol Lett 210:227–232. 10.1111/j.1574-6968.2002.tb11185.x 12044679 10.1111/j.1574-6968.2002.tb11185.x

[CR67] Souza HML, Taniguchi S, Bícego MC, Oliveira LA, Oliveira TCS, Barroso HS, Zanotto SP (2015) Polycyclic aromatic hydrocarbons in superficial sediments of the Negro river in the Amazon region of Brazil. J Braz Chem Soc 26:1438–1449. 10.5935/0103-5053.20150112

[CR68] Strotmann U, Flores DP, Konrad O, Gendig C (2020) Bacterial toxicity testing: modification and evaluation of the luminescent bacteria test and the respiration inhibition test. Processes 8:1–18. 10.3390/pr8111349

[CR69] Teerapatsakul C, Pothiratana C, Chitradon L, Thachepan S (2016) Biodegradation of polycyclic aromatic hydrocarbons by a thermotolerant white rot fungus *Trametes polyzona* RYNF13. J Gen Appl Microbiol 62:303–312. 10.2323/jgam.2016.06.001 27885193 10.2323/jgam.2016.06.001

[CR70] Teh ZC, Hadibarata T (2014) Enhanced degradation of pyrene and metabolite identification by *Pleurotus eryngii* F032. Water Air Soil Pollut 225:1–8. 10.1007/s11270-014-1909-x

[CR71] Terzi E, Samara C (2004) Gas-particle partitioning of polycyclic aromatic hydrocarbons in urban, adjacent coastal, and continental background sites of western Greece. Environ Sci Technol 38:4973–4978. 10.1021/es040042d 15506188 10.1021/es040042d

[CR72] Turek A, Wieczorek K, Wolf WM (2019) Digestion procedure and determination of heavy metals in sewage sludge-an analytical problem. Sustainability 11:4–10. 10.3390/su11061753

[CR73] Valiante V (2017) The cell wall integrity signaling pathway and its involvement in secondary metabolite production. J Fungi 3:68. 10.3390/jof3040068 10.3390/jof3040068 PMC5753170 29371582

[CR74] Vipotnik Z, Michelin M, Tavares T (2021) Ligninolytic enzymes production during polycyclic aromatic hydrocarbons degradation: effect of soil pH, soil amendments and fungal co-cultivation. Biodegradation 32:193–215. 10.1007/S10532-021-09933-2 33725325 10.1007/s10532-021-09933-2

[CR75] Wilcke W, Bandowe BAM, Lueso MG, Ruppenthal M, del Valle H, Oelmann Y (2014) Polycyclic aromatic hydrocarbons (PAHs) and their polar derivatives (oxygenated PAHs, azaarenes) in soils along a climosequence in Argentina. Sci Total Environ 473–474:317–325. 10.1016/j.scitotenv.2013.12.037 24374593 10.1016/j.scitotenv.2013.12.037

[CR76] Woo OT, Chung WK, Wong KH, Chow AT, Wong PK (2009) Photocatalytic oxidation of polycyclic aromatic hydrocarbons: intermediates identification and toxicity testing. J Hazard Mater 168:1192–1199. 10.1016/j.jhazmat.2009.02.170 19361920 10.1016/j.jhazmat.2009.02.170

[CR77] Wu M, Xu Y, Ding W, Li Y, Xu H (2016) Mycoremediation of manganese and phenanthrene by *Pleurotus eryngii* mycelium enhanced by Tween 80 and saponin. Appl Microbiol Biotechnol 100:7249–7261. 10.1007/S00253-016-7551-3 27102128 10.1007/s00253-016-7551-3

[CR78] Yang SYN, Connell DW, Hawker DW, Kayal SI (1991) Polycyclic aromatic hydrocarbons in air, soil and vegetation in the vicinity of an urban roadway. Sci Total Environ 102:229–240. 10.1016/0048-9697(91)90317-8

[CR79] Ye J-s, Yin H, Qiang J, Peng H, Qin H-m (2011) Biodegradation of anthracene by *Aspergillus fumigatus*. J Hazard Mater 185:174–181. 10.1016/j.jhazmat.2010.09.015 20932640 10.1016/j.jhazmat.2010.09.015

[CR80] Zafra G, Moreno-Montaño A, Absalón ÁE, Cortés-Espinosa DV (2015) Degradation of polycyclic aromatic hydrocarbons in soil by a tolerant strain of *Trichoderma asperellum*. Environ Sci Pollut Res 22:1034–1042. 10.1007/s11356-014-3357-y 10.1007/s11356-014-3357-y 25106516

[CR81] Zámocký M, Tafer H, Chovanová K, Lopandic K, Kamlárová A, Obinger C (2016) Genome sequence of the filamentous soil fungus *Chaetomium cochliodes* reveals abundance of genes for heme enzymes from all peroxidase and catalase superfamilies. BMC Genomics 17:1–15. 10.1186/s12864-016-3111-6 27681232 10.1186/s12864-016-3111-6 PMC5041501

[CR82] Zeiner CA, Purvine SO, Zink EM, Paša-Tolić L, Chaput DL, Haridas S, Wu S, LaButti K, Grigoriev IV, Henrissat B (2016) Comparative analysis of secretome profiles of manganese (II)-oxidizing Ascomycete fungi. PLoS ONE 11:e0157844. 10.1371/journal.pone.0157844 27434633 10.1371/journal.pone.0157844 PMC4951024

[CR83] Zhang S, Ning Y, Zhang X, Zhao Y, Yang X, Wu K, Yang S, La G, Sun X, Li X (2015) Contrasting characteristics of anthracene and pyrene degradation by wood rot fungus *Pycnoporus sanguineus* H1. Int J Biol Macromol 105:228–232. 10.1016/j.ibiod.2015.09.012

