# Supplementary material for: Mycotransformation of anthracene by indigenous Trichoderma lixii and Talaromyces pinophilus isolates: insights into the metabolic pathways, enzyme profiles and acute toxicity
Source: Biodegradation. 2025 Jun 18;36(4):51. doi: 10.1007/s10532-025-10147-z (PMC12176947; doi:10.1007/s10532-025-10147-z)
Supplement: Supplementary file 1 — Supplementary file1 (PDF 392 KB) [file 10532_2025_10147_MOESM1_ESM.pdf]

Supplementary Materials

Mycotransformation of Anthracene by Indigenous *Trichoderma lixii* and *Talaromyces pinophilus* isolates: Insights into the Metabolic Pathways, Enzyme Profiles and Acute Toxicity

Samson O. Egbewale[1], Ajit Kumar[1], Mduduzi P. Mokoena[2] and Ademola O. Olaniran[1*]

[1]Discipline of Microbiology, University of KwaZulu-Natal (Westville Campus), Durban 4000, South Africa

[2]Department of Pathology, School of Medicine, University of Limpopo, Private Bag X1106, Sovenga-0727, South Africa.

* Corresponding author. Tel. +27 31 260 7400/7401.
Fax: +27 31 260 7809
Email address: olanirana@ukzn.ac.za

Fig. S1. Anthracene degradation kinetic modelling (A) Zero Order, (B) First Order, (C) Second Order. Key: *Tl*FLU1 (●), *Tp*FLU12 (■), d = days.

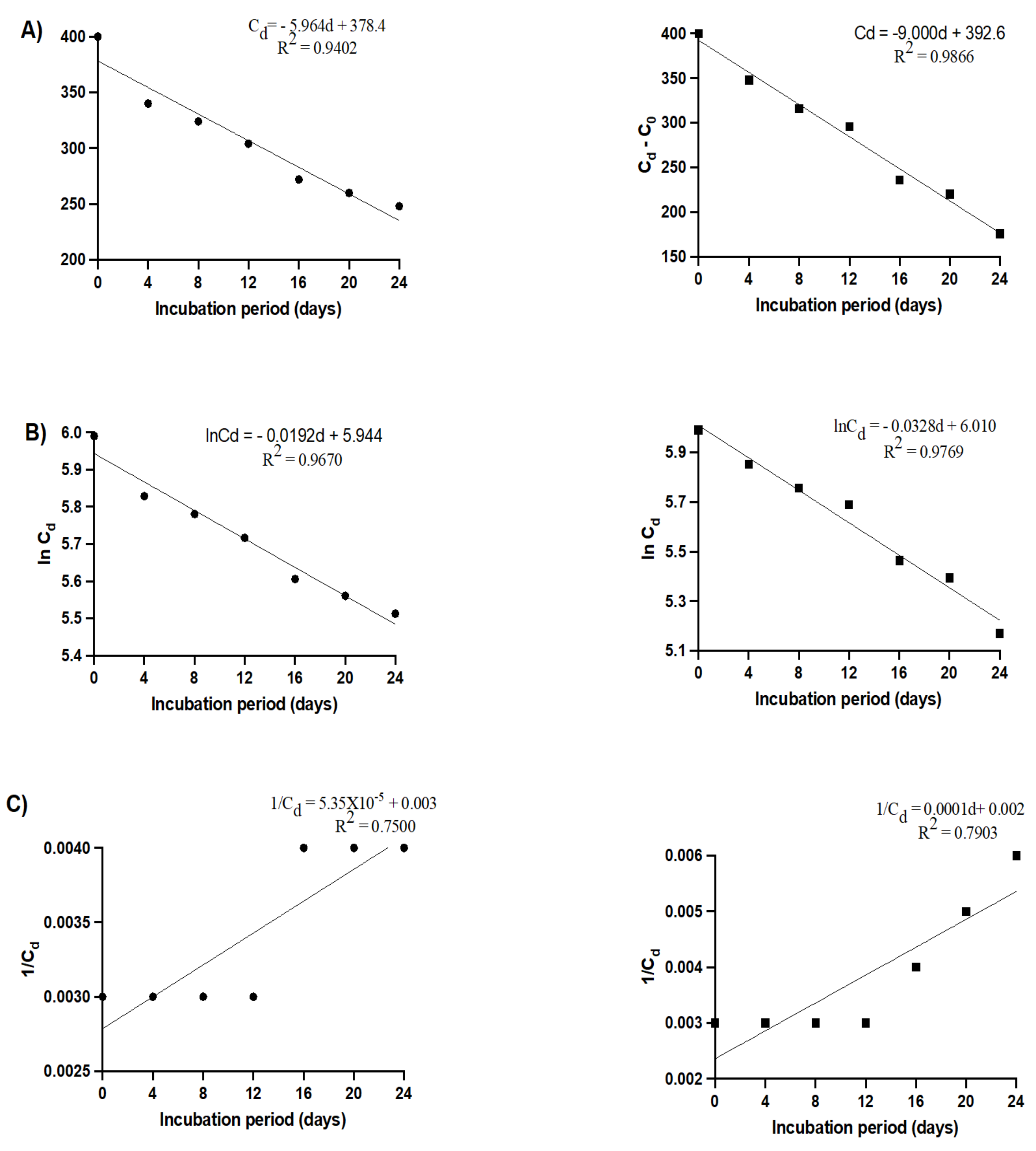

Fig. S2. FTIR spectra of anthracene myco-transformation metabolites.

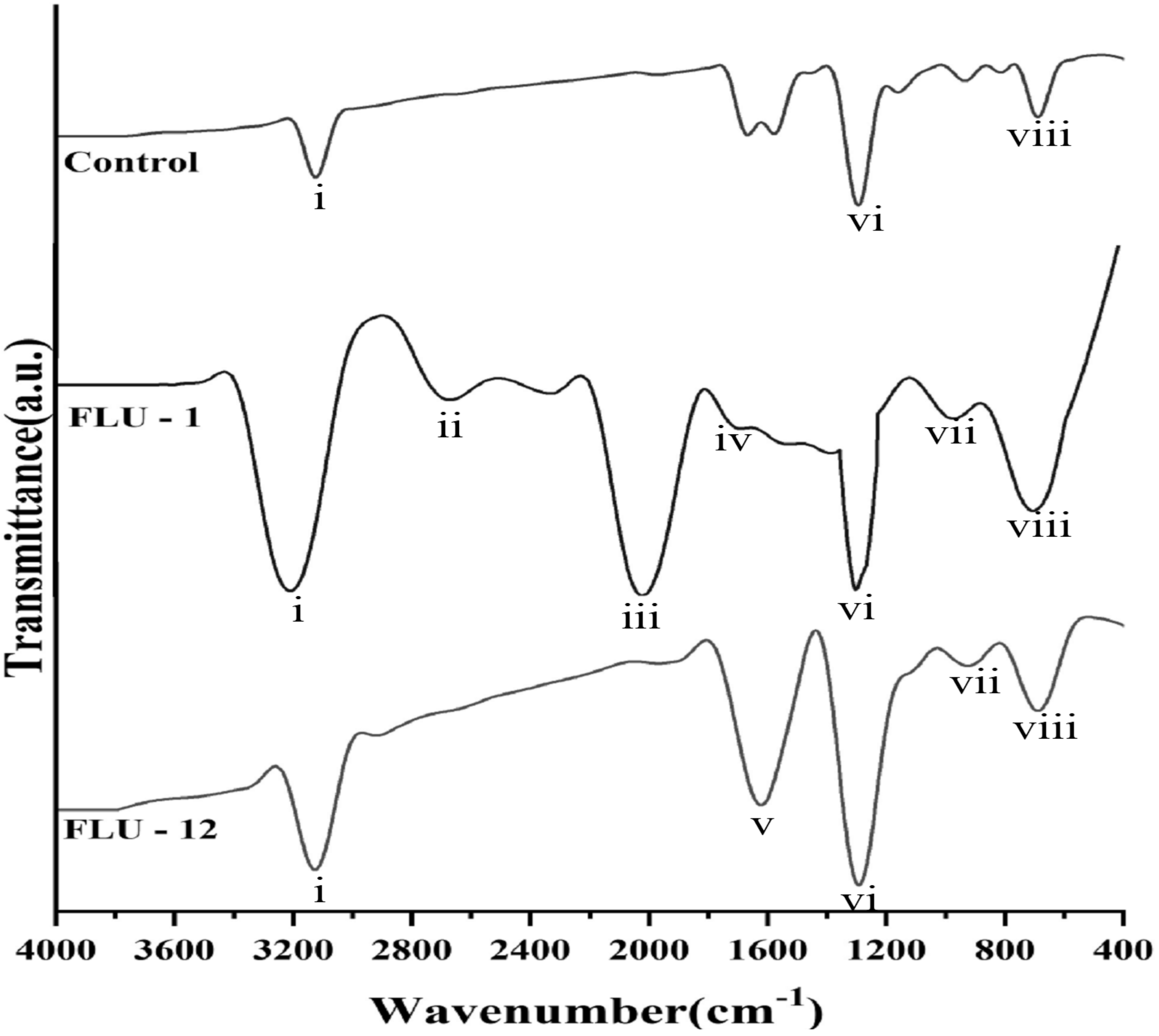

Table S1. Radial extension rate and growth inhibition of fungal strains *Tl*FLU1 and *Tp*FLU12 in response to anthracene concentrations

Concentration	Radial mycelial extension (mm/d)		Growth Inhibition (%)	
(mg/L)	***Tl*FLU1**	***Tp*FLU12**	***Tl*FLU1**	***Tp*FLU12**
0	4.50 ± 0.787[a]	4.40 ± 0.719[a]	0	0
50	4.50 ± 1.035[a]	4.40 ± 1.154[a]	0	0
100	4.50 ± 1.069[a]	4.40 ± 1.175[a]	0	0
200	4.50 ± 0.805[a]	4.39 ± 0.189[a]	0	0
400	4.49 ± 0.432[a]	4.30 ± 0.303[a]	0	2.27 ± 0.381[d]
600	3.80 ± 0.361[a]	3.70 ± 0.830[a]	15.6 ± 0.499[c]	15.9 ± 0.436[c]
800	3.10 ± 0.771[a]	3.50 ± 1.011[a]	31.1 ± 0.023[b]	20.5 ± 0.252[b]
1000	2.70 ± 0.027[a]	3.30 ± 0.642[a]	40.0 ± 1.079[a]	25.0 ± 0.0252[a]

Each value is the means± standard error of four replicates. Values in the same column with different letters as superscripts are significantly different ($p \leq$ _0.05). The radial mycelial extension rate (mm/d) and growth inhibition of fungal strains *Tl*FLU-1 and *Tp*FLU12 in response to varying anthracene concentrations are shown in Table 1. The results revealed distinct responses to anthracene exposure between both strains. For strain *Tl*FLU-1, the highest radial mycelial extension rate of 4.50 mm/d was recorded at anthracene concentrations of 0 to 400 mg/L. However, at higher concentrations (600, 800 and 1000 mg/L), the radial mycelia rate decreased significantly to 3.80, 3.10 and 2.70 mm/d, respectively, when compared to the control. Conversely, strain *Tp*FLU-12 reveals a different pattern. The highest radial mycelial extension rate of 4.40 mm/d was recorded in concentrations 0 – 200 mg/L, while a gradual decrease in radial mycelia rate of 4.30, 3.70, 3.50 and 3.30 mm/d were recorded at concentrations 400, 600, 800 and 1000 mg/L respectively. Similar to *Tl*FLU1, these results indicate a significant reduction compared to the control as anthracene concentration increased. Also, no significant differences were observed among the radial mycelial extension rates at concentrations 0 – 100 mg/L at $p \leq 0.05$ for strains *Tl*FLU1 and *Tp*FLU-12 respectively. Furthermore, the growth inhibition percentages differed between the two strains. *Tl*FLU1 exhibited the highest inhibition at 40%, while *Tp*FLU12 showed a maximum inhibition of 25%. Interestingly, *Tl*FLU1 showed no growth inhibition at concentrations ranging from 0 to 400 mg/L, whereas *Tp*FLU12 only exhibited 0% growth inhibition at concentrations ranging from 0 to 200 mg/L.

Table S2. Qualitative assay for Ligninolytic enzyme production (mm).

Concentration (mg/L)	Oxidation for laccase production		Oxidation for lignin peroxidase production		Oxidation for manganese peroxidase activities	
	***Tl*FLU1**	***Tp*FLU12**	***Tl*FLU1**	***Tp*FLU12**	***Tl*FLU1**	***Tp*FLU12**
0	58. 5 ± 0.761^{bc}	52.8 ± 2.107^{c}	40.9 ± 4.961^{bc}	15.8 ± 3.780^{a}	5.871 ± 11.18^{b}	42.3 ± 8.310^{c}
50	90.0 ± 10.71^{a}	88.0 ± 0.856^{a}	63.0 ± 1.641^{a}	26.4 ± 12.08^{a}	27.0 ± 6.883^{a}	70.4 ± 1.804^{a}
100	90.0 ± 2.720^{a}	88.0 ± 11.21^{a}	63.0 ± 0.660^{a}	26.4 ± 7.863^{a}	27.0 ± 11.43^{a}	70.4 ± 3.150^{a}
200	90.0 ± 0.891^{a}	88.0 ± 0.322^{a}	63.0 ± 2.450^{a}	26.4 ± 6.571^{a}	27.0 ± 0.666^{a}	70.4 ± 3.890^{a}
400	87.0 ± 2.030^{a}	79.2 ± 7.091^{ab}	60.0 ± 1.704^{a}	25.4 ± 8.037^{a}	32.4 ± 2.018^{a}	63.4 ± 5.66^{ab}
600	67.5 ± 1.311^{b}	66.0 ± 6.930^{bc}	47.2 ± 1.234^{b}	19.8 ± 1.172^{a}	20.3 ± 6.290^{ab}	52.8±12.15^{abc}
800	49.5 ± 3.964^{cd}	57.2 ± 10.66^{c}	34.7 ± 8.950^{c}	17.1 ± 12.09^{a}	14.8 ± 0.808^{ab}	45.8±6.23^{bc}
1000	43.2 ± 0.953^{d}	48.4 ± 10.95^{c}	30.2 ± 6.180^{c}	14.5 ± 11.23^{a}	12.9 ± 8.837^{ab}	38.7±11.35^{c}

Each value is the means±standard error of four replicates. Values in the same column with different letters as superscripts are significantly different ($p \leq 0.05$). The qualitative assay for ligninolytic enzyme production of fungal strains *Tl*FLU1 and *Tp*FLU12 in response to exposure to varying anthracene concentrations is shown in Table S2. For laccase production, *Tl*FLU1 exhibited a maximum oxidation diameter of 90.0 mm recorded for 50 to 200 mg/L anthracene concentration, followed by 400 mg/L anthracene concentration with an oxidation diameter of 87.0 mm, *Tl*FLU1 exhibited an oxidation rate of 43.2 ± 0.954 units while 600 to 1000 mg/L concentrations yielded oxidation diameters of 67.5, 49.5, and 43.2 mm, respectively, compared to the control with a 58.5 mm oxidation diameter. A similar trend was observed for strain *Tp*FLU12, with a maximum oxidation diameter of 88.0 mm at 50 to 200 mg/L, followed by 79.2 mm at 400 mg/L. Concentrations of 600 to 1000 mg/L resulted in oxidation diameters of 66.0, 57.2, and 48.4 mm, respectively, compared to the control with a 58.5 mm oxidation diameter. No significant differences were observed among oxidation diameters of anthracene concentrations of 50 – 400 mg/L for *Tl*FLU1 while *Tp*FLU12 showed no significant difference among oxidation diameters of anthracene concentrations of 50 – 200 mg/L at $p \leq 0.05$. Also, both strains exhibited decreasing oxidation diameters with increasing anthracene concentrations for lignin peroxidase production. *Tl*FLU1 displayed a maximum oxidation diameter of 63.0 mm at anthracene concentrations of 50–200 mg/L, followed by 60.0 mm at 400 mg/L, while concentrations of 600 to 1000 mg/L yielded oxidation diameters of 47.2, 34.7, and 30.2 mm, respectively, compared to the control's 40.9 mm oxidation diameter. Conversely, lignin peroxidase production was lower in *Tp*FLU12 than in *Tl*FLU1. For manganese peroxidase activities, both strains exhibited a decrease in oxidation rates with increasing anthracene concentrations, similar to lignin peroxidase production. However, in contrast to lignin peroxidase in *Tl*FLU1, manganese peroxidase was observed to be highly produced in *Tp*FLU-12, with a maximum oxidation diameter of 70.4 mm at 50–200 mg/L, followed by 63.4 mm at 400 mg/L. Concentrations of 600 to 1000 mg/L resulted in oxidation diameters of 52.8, 45.8, and 38.7 mm, respectively, compared to the control with a 42.3 mm oxidation diameter.
